# A Near-Infrared-II Luminescent and Photoactive Vanadium(II) Complex with a 760 ns Excited State Lifetime

**DOI:** 10.1021/jacs.5c04471

**Published:** 2025-06-03

**Authors:** Alexandra König, Robert Naumann, Christoph Förster, Jan Klett, Katja Heinze

**Affiliations:** Department of Chemistry, 9182 Johannes Gutenberg University Mainz, Duesbergweg 10-14, Mainz 55128, Germany

## Abstract

Ruthenium and iridium are key components in the most important applications of photoactive complexes, namely, light-emitting devices, photocatalysis, bioimaging, biosensing, and photodynamic therapy. Especially, near-infrared (NIR) emissive materials are required in fiber-optic telecommunications, anticounterfeit inks, night-vision readable displays, and bioimaging. Replacing rare and expensive precious metals with more abundant first-row transition metals is of great interest; however, photophysical properties and the chemical stability of 3d metal complexes are often insufficient. Here, we tackle these challenges with a nonprecious metal polypyridine vanadium­(II) complex that shows emission above 1300 nm with excited state lifetimes of up to 760 ns. Strong light absorption in the visible spectral region and exceptional stability in the presence of oxygen enable photocatalysis in water and acetonitrile using green to orange-red light for excitation. This study unravels a new design principle for NIR-II luminescent and photoactive complexes based on the abundant first-row transition metal vanadium.

## Graphical abstract

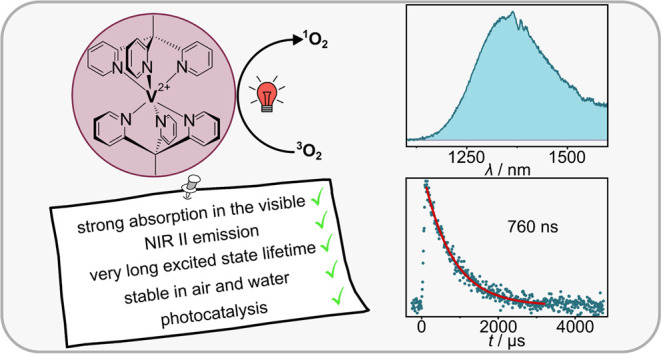

## Introduction

The photophysics and photochemistry of earth-abundant metals^1–6^ are of paramount importance for achieving sustainable light-driven applications, such as light-emitting devices, photocatalysis, bioimaging, and photodynamic therapy,^7–13^ which are dominated by precious metal complexes typically based on ruthenium­(II) or iridium­(III).^14,15^ Complexes possessing metal-to-ligand charge transfer (MLCT),^16,17^ ligand-to-metal charge transfer (LMCT),^18^ and spin-flip (SF)^16,19^ excited states with suitably long excited-state lifetimes and luminescence quantum yields emerged in recent years, which rival the photophysical properties of precious metal complexes.^14,15^ Currently, d^10^-copper­(I),^20–22^ d^6^-molybdenum­(0) (MLCT),^23–27^ d^6^-manganese­(I) (MLCT),^28,29^ d^5^-iron­(III),^30–33^ d^0^-zirconium­(IV) complexes (LMCT),^34,35^ and d^3^‑chromium­(III) complexes (SF)^36–40^ stand at the forefront in particular in photoredox catalytic applications.

Near-infrared (NIR) emissive materials are required for applications in light-emitting devices,^41,42^ fiber-optic telecommunications,^43^ anticounterfeit inks,^44^ night-vision-readable displays,^45^ and in biosensing and bioimaging.^46^ Current NIR luminophores are based on organic dyes,^47–49^ complexes with lanthanide^46,50^ or second- or third-row transition metal elements,^10,11^ or a combination thereof.^51,52^ Chromium­(III) complexes based on an earth-abundant and easily accessible element present a notable exception and typically emit in the red to NIR-I spectral region with long photoluminescence lifetimes and quantum yields of up to 30%.^53–62^

In particular, the NIR-II spectral region (1000–1700 nm) is an important window for medical diagnostics and in vivo imaging due to the deep penetration depth, high spatial resolution, high signal-to-background ratio, low optical absorption and scattering from biological matter, and reduced interfering signals.^49^ While organic NIR-II fluorophores are intrinsically hydrophobic preventing direct application in vivo,^49^ metal complexes can be water-soluble thanks to their (often positive) charge. However, thermal and photochemical (substitutional) stabilities of many metal complexes in water can limit practical applications in water so that water-stable NIR-II emissive complexes are very rare.

While several complex classes have been devised to exhibit red to NIR-I (700–950 nm) luminescence,^53–63^ complexes from earth-abundant metals showing NIR-II luminescence are limited to very few examples (Chart [Fig chart1]), namely, complexes containing d^2^-vanadium­(III) (1100–1256 nm, 293 K and 77 K)^64^−,^65,66,67^ d^3^-chromium­(III) (1067 nm, 77 K),^68^ d^3^-manganese­(IV) (1470 nm, 293 K),^69^ and d^3^-molybdenum­(III) (1250, 1310, 1550 nm, 293 K) (Chart [Fig chart1]).^70^ All these NIR-II luminophores are metal complexes with d^2^ or d^3^ electron configuration capable of displaying either pure SF or CT-admixed SF emission.^19^

**1 chart1:**
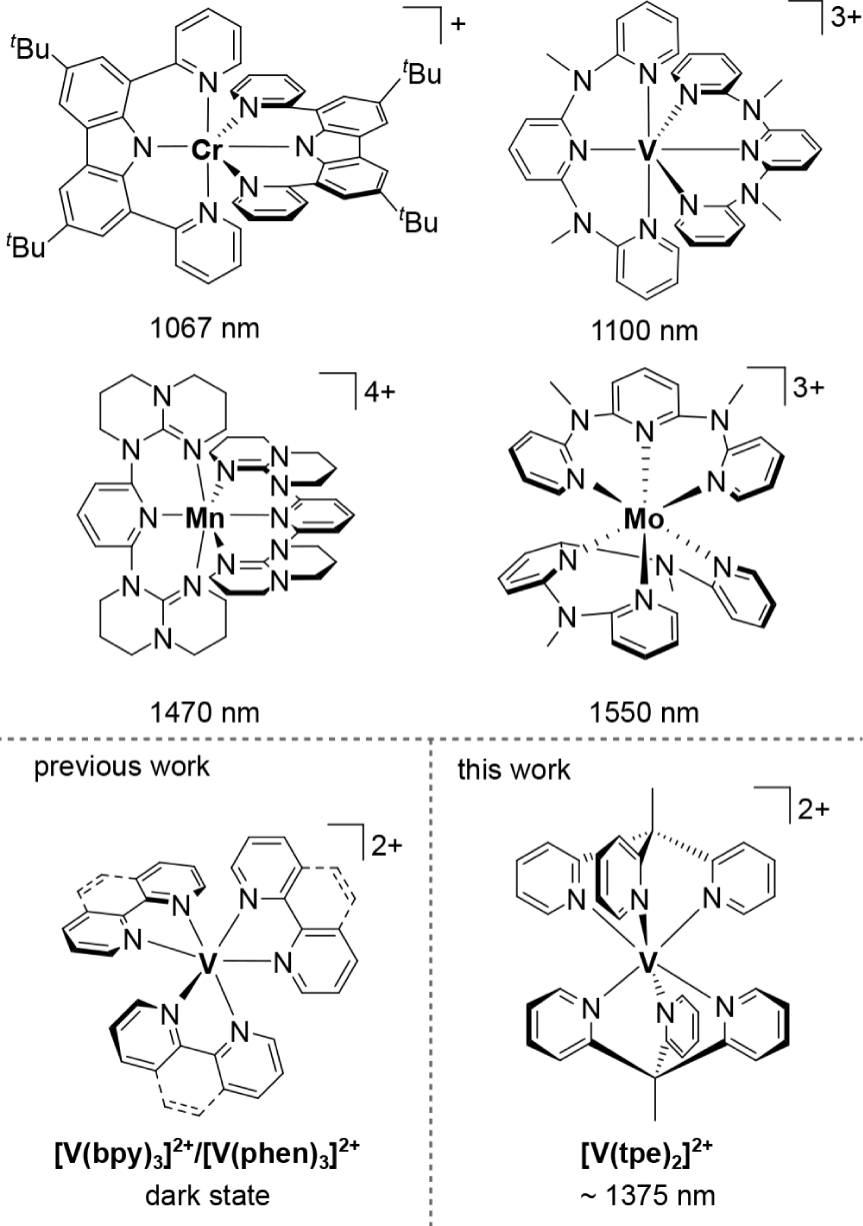
NIR-II Emissive Complexes with Earth-Abundant Metals^a^

a Emission wavelength maxima.

From simple ligand field considerations, the doublet excited states of d^3^-vanadium­(II) ions should possess low energies, potentially in the NIR-II spectral region, thanks to their small free ion Racah parameter *B* of *B*_free_(V_^2+^_) = 766 cm^–1^ as compared to that of chromium­(III) with *B*_free_(Cr_^3+^_) = 914 cm^–1^.^71^ However, the photophysics and photochemistry of d^3^-vanadium­(II)-based materials—although isoelectronic to the well-explored chromium­(III) analogue—are highly underexplored. Merely, vanadium­(II)-doped oxides such as MgO:V^2+^ or Al_2_O_3_:V^2+^ have been reported to display weak SF emission between 855 and 870 nm,^72^ similar to the ruby Al_2_O_3_:Cr^3+^ SF emission at 649 nm,^73^ while luminescent molecular vanadium­(II) complexes have not yet been devised so far.^74–77^ Very short excited-state lifetimes of 0.43 (0.50) ns and 1.6 (1.8) ns in acetonitrile^74^ (ethanol^78^) obtained by transient absorption spectroscopy have been reported for the polypyridine complexes [V­(bpy)_3_]^2+^ and [V­(phen)_3_]^2+^, respectively (bpy = 2,2’-bipyridine, phen = 1,10-phenanthroline; Chart [Fig chart1]). However, no luminescence was observed up to 1600 nm.^74^ The nature of their excited doublet states seems to be a mixture of SF and MLCT characters, contrasting the pure SF character of chromium­(III) doublet excited states.^74,75^ MLCT admixture to the SF states should shift the lowest doublet states to even lower energies. Such low energies are notoriously difficult to measure due to the increase of the nonradiative rate, the decrease of the radiative rate with lower energy, and the lower sensitivity of NIR radiation detection.^79–81^

Vanadium­(II) ions in water have been reported to reduce protons to dihydrogen by high-energy UV-B light irradiation (313 nm).^82^ With a suitable electron acceptor such as methyl viologen, [V­(phen)_3_]^2+^ is initially photooxidized to the vanadium­(III) complex [V­(phen)_3_]^3+^ at pH 8. Subsequently, [V­(phen)_3_]^3+^ dissociates a phen ligand and dimerizes via hydroxido ligands to give {(μ–OH)_2_[V­(phen)_2_]_2_}^4+^.^83^ The latter is further oxidized to vanadyl species [VO]^2+^ so that an irreversible vanadium­(II) to vanadium­(IV) two-electron oxidation process is achieved.^83^ Stoichiometric and catalytic pinacol couplings using vanadium­(II) as the reducing agent have been reported.^84,85^ In a catalytic cycle, a 2,2’-bipyridine chlorido vanadium­(II) intermediate has been suggested, but the photophysics of this four-coordinate species was not reported.^85^ So clearly, the redox chemistry,^86,87^ photophysics, photostability, and photochemistry of potentially NIR-II-emissive and water-soluble vanadium­(II) complexes are insufficiently understood and explored.

Here, we present a water-soluble, water-stable, and photostable polypyridine vanadium­(II) complex **[V­(tpe)**_**2**_]^**2+**^ (Chart [Fig chart1]) that strongly absorbs visible light up to the red spectral region and emits in the NIR-II spectral region (tpe = 1,1,1-tris­(pyrid-2-yl)­ethane).^55,88^ The excited state energy and excited state lifetime of several hundred nanoseconds suffice to form singlet oxygen under air, which is exploited in green (560 nm) and orange-red (625 nm) light-driven photocatalysis in both water and in acetonitrile.

## Results and Discussion

### Synthesis, Structure, and Ground State Reactivity of [V­(tpe)_2_]^2+^

Two equivalents of the tripodal ligand tpe^88^ were coordinated to vanadium­(II) in a microwave-assisted reaction of tpe and [V­(CH_3_CN)_6_]­[BPh_4_]_2_ ^89^ in CH_3_CN/diglyme to give purple **[V­(tpe)_2_]­[BPh_4_]_2_** in 72% yield. For comparative structural and spectroscopic studies, the [BPh_4_]^−^ counterion was exchanged to X^–^ = Cl^–^, [BF_4_]^−^, and [PF_6_]^−^ using the respective tetra-*n*-butyl ammonium or sodium salts for salt metathesis (Figure 1a).

**Figure 1:**
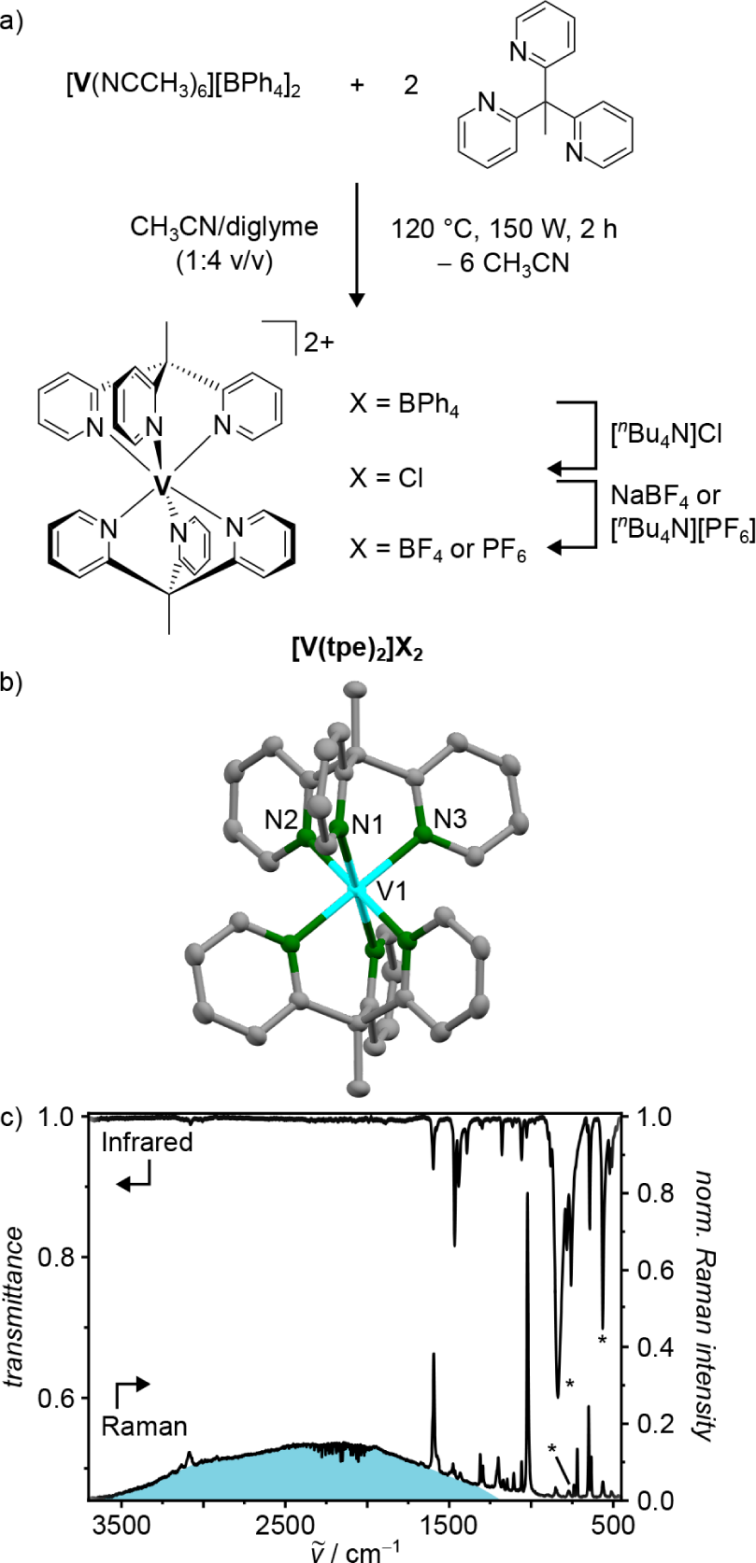
Synthesis of vanadium­(II) complexes **[V­(tpe)_2_]­[X]_2_**. b) Structure of the dication of **[V­(tpe)_2_]­[BPh_4_]_2_** in the solid state with thermal ellipsoids shown at 50% probability. Hydrogen atoms and counterions are omitted. c) IR and Raman spectra of **[V­(tpe)_2_]­[PF_6_]_2_** in the solid state. Asterisks denote bands of the counterion. IR/Raman spectra of other salts **[V­(tpe)_2_]­[X]_2_** as well as DFT calculated vibrational spectra are depicted in Supporting Information, Figure S4.

The composition of the cation is confirmed by ESI^+^ mass spectra showing peaks for **[V­(tpe)**_**2**_]^**2+**^ and {**[V­(tpe)**_**2**_]^**2+**^+X^–^}^+^ (Figure S1). Crystals suitable for single-crystal X-ray analyses were obtained for **[V­(tpe)**_**2**_ **]­[BPh**_**4**_]_**2**_, **[V­(tpe)**_**2**_ **]­[BPh**_**4**_]_**2**_ **×DMF**, **[V­(tpe)**_**2**_ **]­Cl**_**2**_ **×2H**_**2**_ **O×acetone**, **[V­(tpe)**_**2**_ **]­[BF**_**4**_]_**2**_ **×CH**_**3**_ **OH**, and **[V­(tpe)**_**2**_ **]­[PF**_**6**_]_**2**_ **×0.5CH**_**3**_ **OH** (Figure S2 and Table S1, CCDC numbers 2416161–2416165). The molecular structure of the centrosymmetric dication of **[V­(tpe)**_**2**_ **]­[BPh**_**4**_]_**2**_ is depicted in Figure 1b. The metrics of the complex cation are essentially independent of the counterions and the presence of solvent molecules. The experimentally determined average V–N distances and the intra- and *trans*-interligand N–V–N angles amount to 2.11 Å and 85°/180°, respectively. These are well reproduced by DFT geometry optimizations on the B3LYP/def2-TZVPP level of theory (Figure S3, Tables S1 and S2).

The dication of **[V­(tpe)**_**2**_ **]­X**_**2**_ displays only a few vibrational bands in the solid-state IR and Raman spectra due to its high symmetry (Figure S4). The vibrational spectra of **[V­(tpe)**_**2**_ **]­[PF**_**6**_]_**2**_ are exemplarily depicted in Figure 1c. Vibrational frequencies obtained from DFT frequency calculations on **[V­(tpe)**_**2**_]^**2+**^ match the experimentally determined values very well, and bands derived from counterions are easily identified (Figure S4). In addition to the sharp Raman bands obtained after excitation with 1064 nm (9400 cm^–1^), all complex salts investigated display a broad band spanning the region from 3500 to 1200 cm^–1^ (Figure 1c and S4). This band pertains to the cation **[V­(tpe)**_**2**_]^**2+**^ as the band appears independent of the counterion X^–^ in all Raman spectra of the complexes. This phenomenon will be further discussed in the section on excited state properties.

The purple vanadium­(II) complex is soluble and stable in water, acetone, DMF, CH_3_CN, CH_3_OH, and CH_2_Cl_2_. The UV/vis absorption spectrum of **[V­(tpe)**_**2**_ **]­Cl**_**2**_ in water displays a characteristic strong absorption band pattern in the visible spectral region peaking at 571 nm (ε = 7835 M^–1^ cm^–1^) accounting for the purple color (Figure 2a). These bands are weakly solvatochromic (Figure S5). Time-dependent DFT (TD-DFT, Figure 2a, Table S3 and S6) calculations assign ^4^MLCT character to all relevant transitions in this spectral region with only a small admixture of metal-centered quartet states (^4^MC). The weak Laporte-forbidden metal-centered ligand field transitions ^4^A_2_ → ^4^T_2_ and ^4^A_2_ → ^4^T_1_ (calculated transition numbers 20, 21, 23 and 36, 37, 38) appear at higher energy (Table S3). This suggests a large ligand field splitting of the 3d orbitals in the Franck–Condon geometry. The lowest energy ^4^MLCT transition and a set of four intense nearly degenerate transitions are calculated at 592 nm (2.09 eV) and 587 nm, respectively (shifted to lower energy by 923 cm^–1^) according to the TD-DFT calculation. On the other hand, the MLCT band maxima of [V­(bpy)_3_]^2+^ and [V­(phen)_3_]^2+^ at ca. 650 nm are found at much lower energy (by 0.26 eV) due to the lower energy of the π* orbitals of the conjugated bipyridine ligand structure.^74^

**Figure 2:**
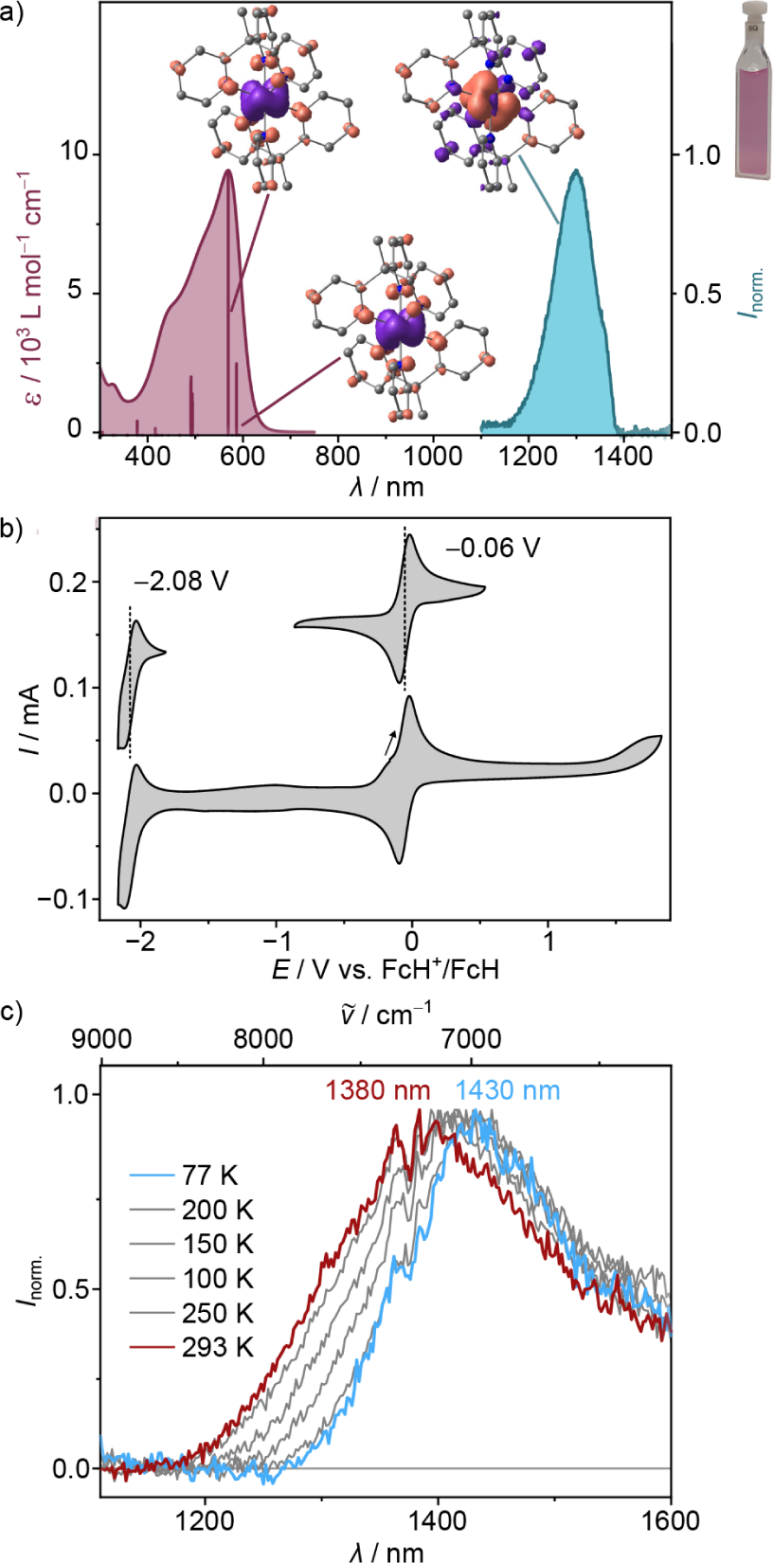
UV/vis absorption and emission spectrum of **[V­(tpe)_2_]­Cl_2_** in H_2_O. TD-DFT calculated transitions (vertical bars, shifted bathochromically by 923 cm^–1^), electron density difference maps [B3LYP/Def2-TZVPP] of optimized **[V­(tpe)_2_]^2+^** showing electron density gain (blue) and depletion (red) in the ^4^MLCT­(5) and ^4^MLCT­(7) Franck–Condon states and spin density map of the optimized lowest energy doublet state (isosurfaces at 0.003 au, H atoms omitted for clarity). Photograph of the aqueous complex solution. b) Cyclic voltammogram of **[V­(tpe)_2_]­[PF_6_]_2_** in CH_3_CN/[^*n*^Bu_4_N]­[PF_6_]. c) Normalized emission spectra of **[V­(tpe)_2_]­[BPh_4_]_2_** in the solid state at temperatures between 293 K (red) and 77 K (blue).

The polypyridine vanadium­(II) cation **[V­(tpe)**_**2**_]^**2+**^ is oxidized to the vanadium­(III) complex at −0.06 V vs ferrocene and reduced to the pyridine radical anion complex at −2.08 V (Figure 2b and S7). Both redox processes are reversible on the spectroelectrochemistry time scale (Figure S7). The reduced complex displays characteristic intense bands at 750 and 860 nm. The electrochemical energy difference amounts to 2.02 eV, which fits the optical energy gap of 2.09 eV. This finding additionally supports the MLCT assignment of the low-energy absorption bands. Compared to the half-wave potential of [V­(phen)_3_]^2+/+^ of *E*_1/2_ = −1.56 V,^83^ the **[V­(tpe)**_**2**_]^**2+/+**^ reduction is much more difficult by 0.52 V due to the higher energy of the pyridine’s π* orbital compared to a phen π* orbital. This also fits the observation that the ^4^MLCT states of **[V­(tpe)**_**2**_]^**2+**^ are located at higher energy.

Compared to the [V­(tpy)_2_]^2+^ and [V­(bpy)_3_]^2+^ oxidations, which lead to ligand dissociation,^87^ the **[V­(tpe)**_**2**_]^**3+/2+**^ redox couple appears very robust. Furthermore, the known mixed aqua polypyridine vanadium­(II) complex [V­(bpy)­(H_2_O)­(tpy)]^2+^ oxidizes easily in water under air to the vanadyl complex [V­(bpy)­(O)­(tpy)]^2+^.^86^ In contrast, the present **[V­(tpe)**_**2**_]^**2+**^ complex is stable in water under ambient conditions for extended periods of time (weeks).

During one crystallization attempt of **[V­(tpe)**_**2**_ **]­[PF**_**6**_]_**2**_ in CH_3_CN under ambient conditions (humid air, daylight), a few blue crystals of **[VO­(** *κ* ^**2**^ **-tpe)­(tpe)]­[PF**_**6**_]_**2**_ **×3CH**_**3**_ **CN** were isolated after several months. In the complex dication **[VO­(** *κ* ^**2**^ **-tpe)­(tpe)]** ^**2+**^, one pyridine donor of a tpe ligand is dissociated to provide the coordination site for an oxido ligand comparable to the situation of the heteroleptic complex [V­(bpy)­(O)­(tpy)]^2+^ (Figure S8 and Table S4).^86^ The V=O bond length of 1.593(3) Å is in a range typical for vanadyl complexes.^90^ A room-temperature X-band cw-EPR spectrum in CH_3_CN shows an octet at *g* = 1.9778 with *A*(^51^V) = 255 MHz (nuclear spin *I*(^51^V) = 7/2; Figure S9) confirming the +IV oxidation state of the vanadium center in **[VO­(** *κ* ^**2**^ **-tpe)­(tpe)]­[PF**_**6**_]_**2**_.^86^ For the parent complex **[V­(tpe)**_**2**_ **]­Cl**_**2**_, an EPR spectrum at 77 K showing the *m*_s_ = −1/2 to *m*_s_ = +1/2 transition with *g*_1,2,3_ = 3.957, 3.841, 1.983 and *A*_1,2,3_(^51^V) = 362, 355, 187 MHz was observed (Figure S9). The oxidation of vanadium­(II) to vanadium­(IV) demonstrates a similar, however, significantly slower, reactivity of the present hexapyridine vanadium­(II) complex toward water/oxygen, thanks to its symmetric closed coordination sphere imposed by the tridentate tripodal ligands. This superior stability paves the way for photonic and photochemical applications in water under air. Furthermore, recovery of vanadium­(III) complexes from vanadyl complexes using pinacol as the reductant has been demonstrated^91^ and other oxygen atom acceptors are also conceivable.^90^

### Excited State Dynamics of [V­(tpe)_2_]^2+^

Laser excitation into the MLCT band pattern of **[V­(tpe)**_**2**_]^**2+**^ at 450 nm in water, acetone, DMF, CH_3_CN, CH_3_OH, or CH_2_Cl_2_ gives a broad emission band peaking in the NIR-II spectral region above 1300 nm (Figures S10 and S11). Figure 2a exemplarily shows the experimental emission spectrum of **[V­(tpe)**_**2**_ **]­Cl**_**2**_ in water. As seen from the solvent absorption spectra in this low-energy region, solvent OH/CH overtone and combination absorption bands^92^ mask the true NIR-II emission maxima in the experimental spectra (Figures S10 and S11). Hence, we additionally measured the NIR-II luminescence of the title complex in several deuterated solvents where the OD/CD overtones appear at different energies (Figures S10 and S11). This provides an estimated band maximum around λ_em_ = 1375 nm. With this NIR-II emission, the present complex is one of the very rare transition metal complexes showing emission in this low-energy spectral region (Chart [Fig chart1]).^64–70^

Interestingly, the broad band in the Raman spectra detected at ca. 2200 cm^–1^ (Figure 1c and S4) after excitation with 1064 nm (9400 cm^–1^) corresponding to an energy of (9400 – 2200 = 7200) cm^–1^ very well matches the emission band observed after ^4^MLCT excitation with higher energy light (450 nm, 22,200 cm^–1^; λ_em_ = 1375 nm; 7273 cm^–1^). Hence, apart from the expected Raman scattering, the sample shows weak luminescence after low-energy excitation. This suggests that dark electronic states are present in the energy region around 9400 cm^–1^. These dark states likely possess doublet multiplicity and considerable ^2^MC character so that the multiplicity selection rule and Laporte’s rule severely forbid the transitions to these states. Hence, Raman scattering becomes competitive to the electronic excitation so that both Raman scattering and phosphorescence can be observed simultaneously in the Raman spectra (Figure 1c and S4).

Under argon at 293 K, the luminescence lifetimes of the final, relaxed doublet excited state of **[V­(tpe)**_**2**_ **]­Cl**_**2**_ amount to 760 and 560 ns in CD_3_CN and D_2_O, respectively (Figure S12). We confirmed these high photoluminescence lifetimes by ns-transient absorption spectroscopy in CD_3_CN and D_2_O (Figure S12). The lowest excited doublet state has mixed ^2^MC/^2^MLCT character according to the DFT-calculated spin density distribution and the excited state geometry with slightly contracted V–N bonds and expanded pyridine C–N bonds (Figure S3 and Table S2). The shortened V–N bonds suggest some ^2^MLCT character of this lowest state, as the formally oxidized metal center should favor shorter V–N bonds. Population of π* orbitals of the pyridines, as suggested by the admixed ^2^MLCT character, accounts for expanded pyridine C–N bonds.

Compared with [V­(bpy)_3_]^2+^ and [V­(phen)_3_]^2+^ with their low-energy π* orbitals of the conjugated pyridines, the energy of the hypothetical pure ^2^MLCT states of **[V­(tpe)**_**2**_]^**2+**^ should be much higher. With reference to the ^4^MLCT levels, the energy difference amounts to ca. 0.26 eV. Hence, the ^2^MLCT admixture to the ^2^MC states, which lowers their energy, is less pronounced in **[V­(tpe)**_**2**_]^**2+**^ compared to [V­(bpy)_3_]^2+^ and [V­(phen)_3_]^2+^. Consequently, the ^2^MC/^2^MLCT state energy of **[V­(tpe)**_**2**_]^**2+**^ shifts into a spectral region that is amenable to detection by suitable NIR detectors.

Upon cooling to 77 K in the solid state, the NIR-II emission band sharpens and the maximum shifts from 1380 to 1430 nm to lower energy (Figure 2c). This suggests that a higher-energy excited doublet state can be reached at room temperature, while at lower temperature, only the lowest-energy doublet state is populated. The energy difference of 250 cm^–1^ is in the range of energy differences of the lowest energy emissive ^2^MC (^2^E and ^2^T_1_) states of chromium­(III) complexes.^53,60,93–97^ Hence, this observation is similar to the situation encountered with phosphorescent isoelectronic d^3^-Cr^III^ complexes emitting from equilibrating ^2^E and ^2^T_1_ ligand field states.^93–97^ For vanadium­(II), both emissive states of ^2^E and ^2^T_1_ character additionally possess some ^2^MLCT admixture.^74,75^ The lowest-energy purely metal-centered doublet state (diabatic state) was estimated at the Franck–Condon geometry by CASSCF­(7,12)-NEVPT2 calculations as 10,600 cm^–1^ (Figure S13), which is significantly higher than the experimental emission energy (Figures S10 and S11). The ^2^MLCT mixing to the metal-centered states accounts for the much lower experimental energy. The fwhm of the low-energy NIR emission band of 810 cm^–1^ at 77 K is broader than for pure SF emitters^53–62,96,97^ and agrees with admixed ^2^MLCT character of the emissive doublet state due to the MLCT-induced excited-state distortion (see above).

The ultrafast dynamics of **[V­(tpe)**_**2**_]^**2+**^ in D_2_O after excitation with 570 nm pulses was probed by femtosecond transient absorption spectroscopy (Figure 3 and Figure S14). Analysis of the time-resolved spectral data delivered three time constants τ_1,2,3_ = 530 fs, 1.5 ps, and 122 ps. We tentatively assign these to intersystem crossing (ISC, τ_1_) from the initially populated ^4^MLCT Franck–Condon state(s) to higher adiabatic mixed ^2^MLCT/^2^MC state(s), internal conversion (IC, τ_2_) to the long-lived lower adiabatic mixed ^2^E/^2^MLCT and ^2^T_1_/^2^MLCT states, and localization and vibrational cooling (VC, τ_3_), respectively. The state-to-state evolution to an excited state with different electronic character is clearly evident from the rise of excited state absorption (ESA) bands around 690 nm and above 900 nm and the change of the ground state bleach around 500 nm after 500 fs (Figure 3a, green spectrum to blue spectrum). The low-energy bands are reminiscent of the absorption bands of the reduced complex suggesting MLCT character (Figure S7). The 690 nm band rises and finally decays supporting the excited-state evolution model (Figure S14c). The observed spectral changes also fit reasonably well to TD-DFT-calculated spectra of excited quartet and doublet states (Figure S15).

**Figure 3:**
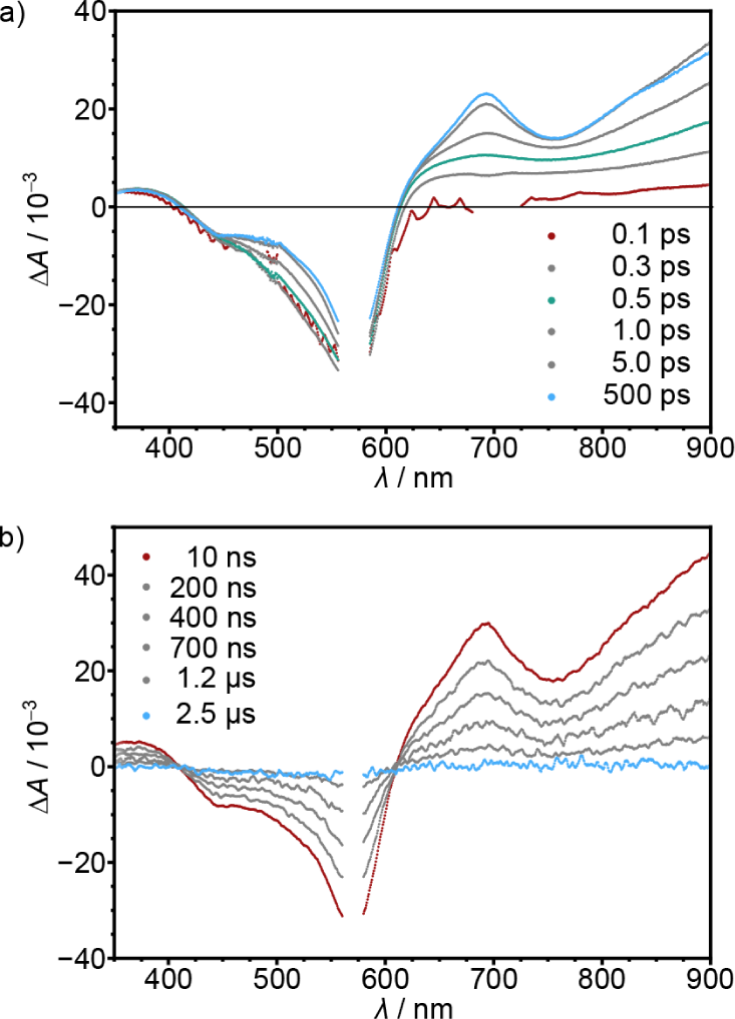
fs-Transient absorption spectra of **[V­(tpe)_2_]­Cl_2_** in D_2_O after excitation with 570 nm at 293 K. b) ns-transient absorption spectra of **[V­(tpe)_2_]­Cl_2_** in D_2_O after excitation with 570 nm at 293 K.

This sequence ^4^A_2_ → ^4^MLCT → ^2^MLCT/^2^MC → ^2^E/^2^MLCT|^2^T_1_/^2^MLCT is schematically illustrated in the qualitative potential energy diagram in Figure 4. The dual emission from the ^2^E/^2^MLCT and ^2^T_1_/^2^MLCT states at room temperature is indicated by two vertical blue downward arrows. This scheme also depicts the direct excitation of the spin-forbidden ^2^MLCT-admixed ^2^E/^2^T_1_ states ^4^A_2_ → ^2^E/^2^MLCT|^2^T_1_/^2^MLCT with 9400 cm^–1^ followed by emission from ^2^E/^2^MLCT and ^2^T_1_/^2^MLCT states, which was observed in the Raman experiments (Figure 1c and S4).

**Figure 4:**
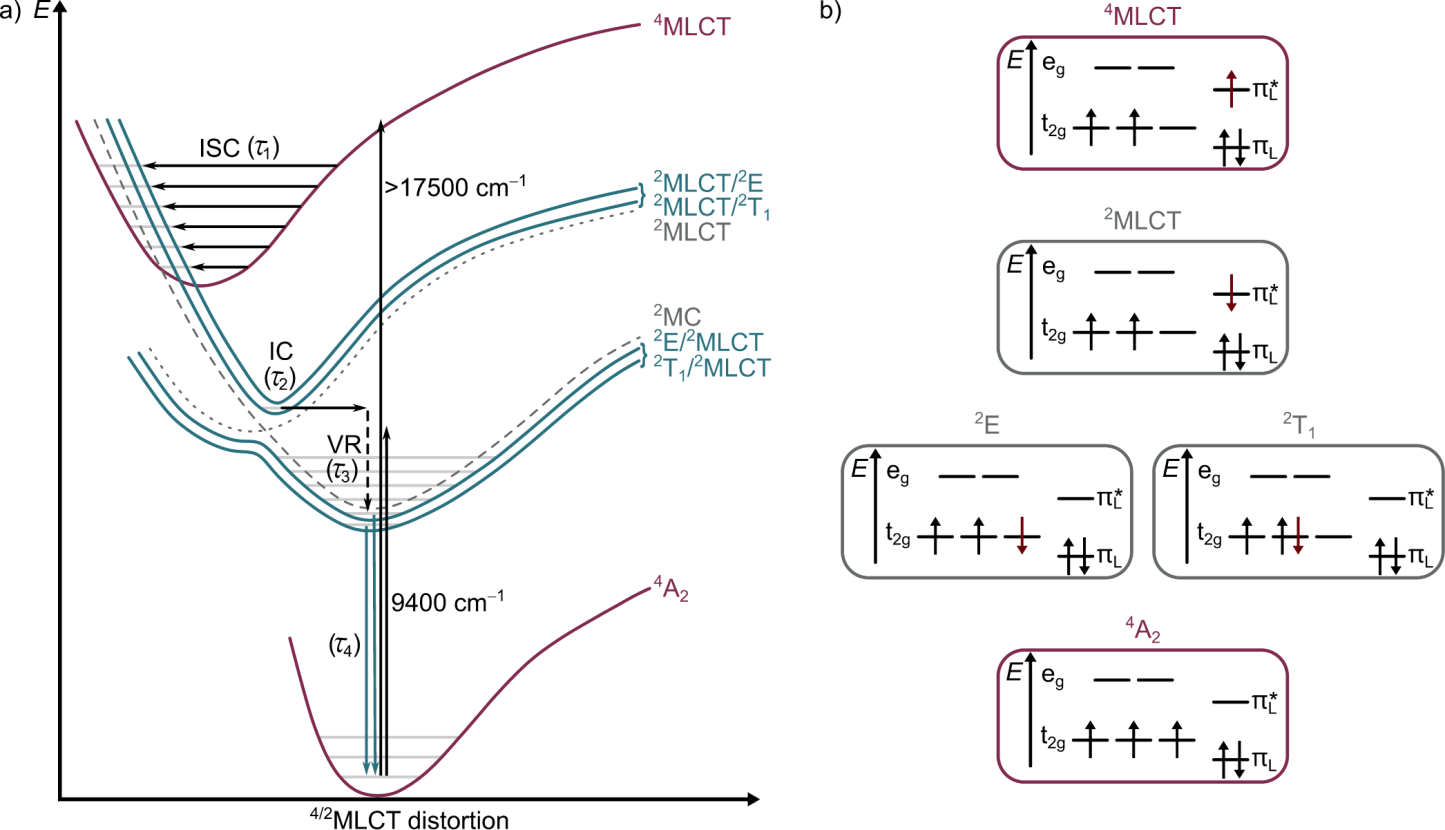
Qualitative scheme of the excited state landscape showing the ^4^A_2_ ground state (purple), the ^4^MLCT excited state (purple) and the adiabatic ^2^MC/^2^MLCT potentials (blue). Exemplary diabatic doublet potentials of hypothetical pure ^2^MC and ^2^MLCT states are shown to illustrate the doublet state mixing (dashed gray). At small-amplitude distortions in the Franck–Condon region, the character is largely metal-centered (^2^E/^2^T_1_) while the adiabatic state gains more ^2^MLCT character at larger distortions. At the adiabatic energy minima, the two lowest-energy emissive states possess mixed ^2^E/^2^MLCT and ^2^T_1_/^2^MLCT character, respectively. b) Exemplary qualitative orbital occupations of the quartet and diabatic doublet states for illustration.

Overall, the long-lived lower-energy emissive ^2^MLCT-admixed ^2^MC states are reached within a few picoseconds after light excitation (Figure 3). Dual emission from these two ^2^MLCT admixed equilibrating ^2^E/^2^T_1_ states is observed at room temperature with a lifetime of several hundred nanoseconds (Figure 2a,c and S12). This long lifetime allows testing of **[V­(tpe)**_**2**_]^**2+**^ as a photosensitizer.

### Excited State Reactivity of [V­(tpe)_2_]^2+^

First, we investigated the photostability of **[V­(tpe)**_**2**_ **]­Cl**_**2**_ in deaerated CH_3_CN and H_2_O with excitation at 450 nm in comparison to the working horse complex [Ru­(bpy)_3_]­Cl_2_ ^14,98^ (Figure S16 and Table S5). The obtained spectroscopic data confirm that **[V­(tpe)**_**2**_ **]­Cl**_**2**_ with photodegradation quantum yields of Φ_deg,V_ = 5.8 × 10^–5^% and 5.0 × 10^–5^% is 590 and 26 times more photostable than [Ru­(bpy)_3_]­Cl_2_ with Φ_deg,Ru_ = 3.4 × 10^–2^ % and 1.3 × 10^–3^ % in acetonitrile and water, respectively. This very favorable stability in both solvents encouraged us to explore the photoreactivity of **[V­(tpe)**_**2**_ **]­Cl**_**2**_ toward triplet oxygen (from air) in water and in acetonitrile.

The excited state lifetime of **[V­(tpe)**_**2**_ **]­Cl**_**2**_ in air-saturated water decreases to 470 ns according to the luminescence lifetime and ns-transient absorption spectroscopy (Figure S12). In fact, the characteristic ^1^O_2_ emission at 1270 nm^99^ is observed upon irradiating CD_3_CN solutions of **[V­(tpe)**_**2**_ **]­Cl**_**2**_ at 450 nm under air (Figure S17). Energy transfer from the mixed ^2^MC/^2^MLCT states to triplet oxygen is with the energy of singlet oxygen (^1^O_2_) of 0.97 eV thermoneutral to slightly endergonic. This energy match of the excited states suggests the possibility of an excited state energy transfer equilibrium^100^ between oxygen and **[V­(tpe)**_**2**_]^**2+**^. In fact, the excited state lifetime of ^1^O_2_ in CD_3_CN reduces from 1.4 ms (unquenched) to 12 μs due to the excited state equilibrium with the vanadium complex (Figure S17). In addition, the vanadium complex shows biexponential decay kinetics in CD_3_CN under air confirming the equilibrium (Figure S12). Kinetic details of the excited state equilibrium in air-saturated acetonitrile are summarized in Figure S17 (kinetic modeling). Due to the low concentration of ^3^O_2_ and the short lifetime of ^1^O_2_ in water, a biexponential kinetics is not observed in air-saturated water (Figure S12).^101^ As ^1^O_2_ forms both in water and in acetonitrile, we tested oxidation reactions of ^1^O_2_ generated from air, **[V­(tpe)**_**2**_]^2+^, and light in both solvents. In the absence of substrates, the formed ^1^O_2_ reacts with the photosensitizer **[V­(tpe)**_**2**_]^**2+**^ in water according to UV/vis-spectroscopic analysis (Figure S16 and Table S5). However, in the presence of substrates, **[V­(tpe)**_**2**_]^**2+**^ is photostable (see below).

We employed **[V­(tpe)**_**2**_ **]­Cl**_**2**_ (0.1 mM), air, and 560 nm light in buffered water as a green and sustainable photooxidizing system. The platform chemical 5-(hydroxymethyl)­furan-2-carbaldehyde (5-HMF), which can be obtained from sugars,^102,103^ was cleanly converted to (*Z*)-5-hydroxy-4-keto-2-pentenoic acid^104^ and the valuable C_1_ building block formate in a 1:1 ratio (Scheme 1a and Figure S18). The C_5_ carboxylic acid formed under the buffered conditions by ring-opening of the initially produced lactone can be accumulated at lower temperature (278 K) and is a valuable potential biobased polyester precursor.^105^ The fact that this photoreaction is successful even at 278 K suggests that strong thermal activation of doublet-singlet energy transfer is unnecessary. The similar excited state energies of O_2_ and **[V­(tpe)**_**2**_]^**2+**^ are also seen from the emission spectra (Figure S17). Gratifyingly, UV/vis monitoring confirmed the high stability of the vanadium-based photosensitizer under these oxidizing and aqueous conditions (Figure S19).

**Scheme 1:**
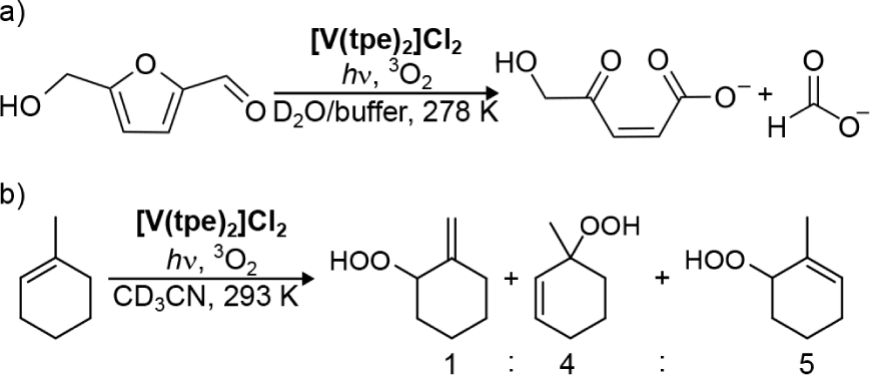
Reactions of (a) 5-HMF and (b) 1-MCH with ^1^O_2_ Formed by **[V­(tpe)**_**2**_]^**2+**^, ^3^O_2_ (Continuous Bubbling of Air through the Solution), and 560 or 625 nm Light in Water or Acetonitrile, Respectively

We further tested singlet oxygen formation in acetonitrile in a ^1^O_2_–based Schenck ene^106^ reaction at room temperature. 1-Methylcyclohexene (1-MCH) is oxidized by ^1^O_2_ to the three different hydroperoxides: 3-hydroperoxy-3-methylcyclohex-1-ene, 6-hydroperoxy-1-methylcyclohex-1-ene, and 1-hydroperoxy-2-methylenecyclohexane.^107^ **[V­(tpe)**_**2**_ **]­Cl**_**2**_ (0.1 mM), air, and green light (560 nm) transform 1-MCH quantitatively to the three hydroperoxide products in a 5:4:1 ratio within 60 min (Scheme 1b and Figure S20).

As the complex **[V­(tpe)**_**2**_]^**2+**^ also absorbs in the orange-red spectral region (Figure 2a), we tested orange-red light (625 nm) in the 1-MCH oxidation. The reaction of **[V­(tpe)**_**2**_]^**2+**^ (0.25 mM) is slower but quantitative after 150 min (Figure S20) suggesting sufficient ^1^O_2_ formation even with orange-red light. Again, **[V­(tpe)**_**2**_]^**2+**^ is very stable under the catalysis conditions in acetonitrile as UV/vis absorption spectra before and after catalysis are essentially superimposable (Figure S21).

The high chemical stability and photostability, sufficient singlet oxygen formation, and orange-red light excitation might pave the way for applications of **[V­(tpe)**_**2**_]^**2+**^ in biological environments requiring low-energy light for deeper penetration depths. The fact that **[V­(tpe)**_**2**_ **]­Cl**_**2**_ can produce sufficient ^1^O_2_ with orange-red light makes this complex competitive with the red-light excitation of polypyridine complexes containing the precious metal osmium.^108–110^

The successful photoxidation with oxygen as the terminal oxidant using green or orange-red light in water and in acetonitrile, respectively, confirms the exceptional stability of the vanadium­(II) photosensitizer under these conditions—in its ground state as well as in its excited states.

## Conclusions

Achieving NIR-II photoluminescence is extremely challenging, in particular, with complexes of abundant first-row transition metals. This study presents a significant advancement in this field using the 3d^3^ metal complex **[V­(tpe)**_**2**_]^**2+**^ with optimally tuned π* energies of the ligand. This allows weak mixing of ^2^MLCT states with the metal-centered spin-flip states so that the phosphorescence emission attains some MLCT character and occurs in the measurable NIR-II spectral region. The excited state lifetime of **[V­(tpe)**_**2**_]^**2+**^ is with 760 ns among the highest reported for 3d transition metal complexes beyond classic chromium­(III) and a few copper­(I) complexes. The long lifetime and high stability imposed by the tripodal ligands enables photosensitization of oxygen in water and acetonitrile using green or orange-red light excitation of the vanadium­(II) complex. The formed singlet oxygen can be employed in typical organic photooxidations such as the Schenck reaction.

Key to success is the tripodal ligand coordinated to the vanadium­(II) ion, ensuring strong visible absorption thanks to MLCT states and chemical stability, imposing a sufficiently large ligand field, and enabling weak mixing of MLCT with spin-flip states. This synthetically very simple, highly colored, NIR-II emissive, water, and oxygen stable first-row transition metal complex also promises future applications in biological settings such as (time-gated) bioimaging with NIR-II light detection or photodynamic therapy.

## Supplementary Material





## Notes

Details of quantum chemical calculations, Cartesian coordinates of optimized geometries, synthetic procedures, analytical and spectroscopic data of **[V­(tpe)**_**2**_ **]­[X]**_**2**_ (PDF)
Cartesian coordinates of optimized geometries (XYZ)
