# Supplementary material for: A Near-Infrared-II Luminescent and Photoactive Vanadium(II) Complex with a 760 ns Excited State Lifetime
Source: J Am Chem Soc. 2025 Jun 3;147(24):20833–42. doi: 10.1021/jacs.5c04471 (PMC12186473; doi:10.1021/jacs.5c04471)
Supplement: Supplementary file 1 [file ja5c04471_si_001.pdf]

A near-infrared-II luminescent and photoactive vanadium(II) complex with a 760 ns excited state lifetime

Alexandra König, Robert Naumann, Christoph Förster, Jan Klett, and Katja Heinze

Department of Chemistry, Johannes Gutenberg University Mainz,
Duesbergweg 10-14, 55128 Mainz, Germany

Supporting Information

General Procedures. All reagents were used as received from commercial suppliers (ABCR, Acros Organics, Alfa Aesar, Fisher, Sigma-Aldrich and TCI). DMF was obtained from VWR, dry diglyme and methanol were purchased from Fisher Scientific. Acetonitrile was distilled over calcium hydride, degassed by the freeze-pump-thaw method and stored over 3 Å molecular sieve. A glovebox (*UniLab*/*MBraun*, Ar 5.0, O_2 < 1 ppm, H_2O < 1 ppm) was used for storage and weighing of sensitive compounds. Dried glassware was used for storage of dry solvents and in the synthesis and recrystallization of the vanadium(II) precursor $[V(NCCH_3)_6][BPh_4]_2$ using standard Schlenk procedures. The ligand tpe and $[V(NCCH_3)_6][BPh_4]_2$ were prepared according to reported procedures.[1,2]

Microwave syntheses were conducted using a *CEM Discover microwave* in a Schlenk flask with pressure compensation. The reaction mixture was prepared in a glovebox.

IR spectra were recorded with a *Bruker Alpha II FTIR spectrometer* with an ATR unit containing a diamond crystal. The intensities are qualitatively indicated with weak (w), medium (m) and strong (s).

Raman spectra were measured on a *Nicolet 5700 FT-IR* spectrometer combined with a *NXR 9650 FT-Raman Module* equipped with a 1064 nm laser (laser power 20–1500 mW; resolution 2 cm^{-1}; number of scans 1024–4098), a Microstage microscope, and a *NXR Genie Ge-detector* using single crystals or crystalline powders in glass capillaries under inert gas. The intensities are qualitatively indicated with weak (w), medium (m) and strong (s).

Electrochemical experiments were carried out on a *BioLogic SP-200* voltammetric analyzer using platinum wires as counter and working electrodes and a 0.01 M Ag/Ag[NO_3] electrode as reference electrode. Cyclic voltammetry and square wave measurements were carried out at scan rates of 50–200 mV s^{-1} using 0.1 M $[^nBu_4N][PF_6]$ in CH_3CN as supporting electrolyte. Potentials are referenced against the ferrocenium/ferrocene couple.

UV/vis/NIR spectroelectrochemical experiments were performed using a *TSC 1600 Spectro cell* from *rhd instruments* equipped with a platinum net working electrode (approximate path length 0.43 mm), a glassy carbon counter electrode and a silver wire as pseudo reference electrode and a *potentiostat Autolab IMP* from *Metrohm*. A *J&M TIDAS S MMS* was used as UV/vis/NIR spectrometer, a *Hamamatsu L10290* as excitation source.

ESI^+ mass spectra were recorded on an *Agilent 6545 QTOF-MS* spectrometer by the central analytical facility of the Department of Chemistry, University of Mainz, Germany.

X-band cw-EPR spectra were measured on a *Miniscope MS300* at 293 K. The *g* factor is referenced to external Mn^{2+} in ZnS (g = 2.118, 2.066, 2.027, 1.986, 1.946, 1.906). The spectrum of **$[VO(\kappa^2\text{-tpe})(tpe)][PF_6]_2$**

was simulated using *EasySpin* (5.2.35) for *Matlab* R2022a using the routine *chili* for cw-EPR spectra of tumbling spin systems in the slow-motional regime, while the spectrum of **[V(tpe)$_2$]Cl$_2$** (measured at 77 K cooled by liquid nitrogen in a finger Dewar (*Magnettech* GmbH, Berlin, Germany)) was simulated using anisotropic fitting.[3]

^{1}H NMR spectra were recorded on a *Bruker Avance II 400* spectrometer at 400 MHz. All resonances are reported in ppm versus the solvent signal as an internal standard [D_2O (^{1}H: δ = 4.79 ppm); CD_3CN (^{1}H: δ = 1.94 ppm)].[4] Multiplicities are abbreviated with singlet (s), doublet (d), triplet (t), and multiplet (m).

Elemental analyses were conducted by the microanalytical laboratory of the department of chemistry of the University of Mainz using an *Elementar vario EL Cube*.

UV/vis/NIR absorption spectra were measured on a *Jasco V770* spectrometer using 1.0 cm quartz cells. Absorption spectra for the photostability tests of the complex were measured on an *Agilent Carry 5000* spectrometer using an air-tight cuvette (d = 1.0 cm).

Steady-state emission spectra and emission decay curves were measured with a *FLS1000 spectrometer* from *Edinburgh Instruments* equipped with a cooled NIR-sensitive photomultiplier detector *N-G09 PMT-1700*. A xenon arc lamp Xe2 (450 W) or a cw-laser *RLTMDL-450-1W-3* (450 nm, P_{max} = 1089 mW) from *Roithner Lasertechnik* was used for excitation in steady-state measurements. Emission decay curves were recorded in the multi-channel scaling mode employing a pulsed diode laser *VPL-450* (λ_{exc} = 451.3 nm) as excitation source. Variable temperature emission measurements were carried out using a liquid nitrogen cooled cryostat *Optistat DN* from *Oxford Instruments*.

fs-Transient absorption experiments were conducted using a *Helios* pump-probe setup from *Ultrafast Systems* paired with a regeneratively amplified 1030 nm laser (Pharos, *Light Conversion*, 1030 nm, <175 fs, 2 mJ). The effective laser repetition rate of 1 kHz was set via an internal pulse picker. A small portion of the 1030 nm fundamental was directed to the optical delay line and was subsequently used to generate broadband probe light by focusing the beam onto a sapphire for measurements in the vis/NIR range (450 – 900 nm). In the UV/vis spectral range (330 – 500 nm), the second harmonic was focused onto a second sapphire. The pump pulse was generated with an optical parametric amplifier (Apollo Y, *Ultrafast Systems*) and the beam diameter at the sample was adjusted to 100 μm at the sample to assure homogeneous excitation of the observation volume, which is defined by the probe diameter (ca. 25 μm). The sample solutions were measured in a 1 mm quartz cuvette. To generate spectra that cover the entire spectral region from 350 nm to 900 nm, the UV/vis and vis/NIR part of the transient absorption spectra were recorded separately under identical conditions and were combined by matching the relative band maxima of both spectral regions with the corresponding ns-transient absorption spectra. For the latter, the entire spectral range can be measured simultaneously. Preprocessing of the data, including chirp and baseline correction, has been performed using the *Surface Xplorer* 4.3.0 software from *Ultrafast Systems*. The open-source *Python* based data analysis tool *KiMoPack* 7.4.9[5] was employed for global analysis of the data.

ns-Transient absorption experiments were carried out using a modified version of the described fs-transient absorption spectroscopy setup. For this purpose, the Eos add-on from *Ultrafast Systems* has been employed, which uses a photonic crystal fiber-based supercontinuum laser as probe light source. In contrast to the fs measurements, the pump-probe time delay is controlled electronically with a time resolution of < 1 ns.

Photostability experiments were conducted by irradiating diluted isoabsorptive (at 450 nm) solutions of **[V(tpe)$_2$]Cl$_2$** and the reference complex [Ru(bpy)$_3$]Cl$_2$ in deaerated H_2O or CH_3CN in air-tight cuvettes (optical path length d = 1 cm) under constant stirring. A cw-laser *RLTMDL-450-1W-3* (450 nm, P_{max} = 1089 mW) with an emission maximum at 450 nm was used for excitation. The temperature was kept constant at 293 K using a Peltier-cooled sample holder. [Ru(bpy)$_3$]Cl$_2$ was irradiated inside the *FLS1000* emission spectrometer, such that its decomposition could be monitored via its characteristic phosphorescence at 620 nm during the irradiation. **[V(tpe)$_2$]Cl$_2$** was also irradiated in the *FLS1000* emission spectrometer. However, due to the weak NIR-II emission of the vanadium complex, the characteristic absorption band was used to monitor the decay. Irradiation has been interrupted at several points in time to measure UV/vis absorption spectra (Fig. S16).

The absolute photodegradation quantum yields $\phi_{deg,x}$ of [Ru(bpy)$_3$]Cl$_2$ and **[V(tpe)$_2$]Cl$_2$** were estimated using equation S1, where $\dot{N_{deg}}$ and $\dot{N_{abs}}$ denote the number of the decomposed complex molecules per unit time and the photon absorption rate, respectively.

$$\phi_{deg,x} = \frac{\dot{N_{deg}}}{\dot{N_{abs}}} \qquad \text{(eq. S1)}$$

$\dot{N_{deg}}$ was calculated using equation S2, with $m_{0,x}$, V and N_A denoting the initial slope of the corresponding concentration time profiles (Fig. S16), the volume of the irradiated solution and Avogadro's constant N_A, respectively.

$$\dot{N_{deg}} = \frac{m_{0,x} \cdot V \cdot N_A}{3.6 \cdot 10^9} \qquad \text{(eq. S2)}$$

$\dot{N_{abs}}$ is given by equation S3, where P_{exc} is the power of the cw-laser (P_{exc} = 0.94 W, determined with a high sensitive thermal power head S401C from *Thorlabs*), λ is the excitation wavelength (λ = 450 nm), h is Planck's constant, c is the speed of light und A is the absorbance at 450 nm.

$$\dot{N_{abs}} = \frac{P_{exc} \cdot \lambda}{h \cdot c}(1 - 10^{-A}) \qquad \text{(eq. S3)}$$

Absolute and relative photodegradation quantum yields ϕ_{rel} ($\phi_{rel} = \frac{\phi_{deg,Ru}}{\phi_{deg,V}}$) are summarized in Table S5.

Crystal Structure Determinations. Intensity data for crystal structure determinations were collected with a *STOE IPDS-2T* diffractometer from *STOE & CIE GmbH* with an *Oxford* cooling using Mo-Kα radiation (λ = 0.71073 Å). The diffraction frames were integrated using the STOE X-Area[6] software package and were corrected for absorption with MULABS[7] of the Platon[8] software package or with STOE X-Red of the STOE X-Area[6] software package. The structures were solved with SHELXT[9] and refined by the full-matrix method based on F^2 using the SHELXL[10] of the SHELX software package[11] and the ShelXle graphical interface.[12] All non-hydrogen atoms were refined anisotropically, while the positions of all hydrogen atoms were generated with appropriate geometric constraints and allowed to ride on their respective parent atoms with fixed isotropic thermal parameters. Crystallographic data for the structures reported in this paper have been deposited with the Cambridge Crystallographic Data Centre as supplementary publication no. 2416161 (**[V(tpe)$_2$][BPh$_4$]$_2$**), 2416162 (**[V(tpe)$_2$][BPh$_4$]$_2$x2DMF),** 2416163 (**[V(tpe)$_2$]Cl$_2$×2H$_2$O×acetone**), 2416164 (**V(tpe)$_2$][BF$_4$]$_2$×MeOH**), 2416165 (**[V(tpe)$_2$][PF$_6$]$_2$×0.5MeOH**) and 2416166 (**[VO(κ^2-tpe)(tpe)][PF$_6$]$_2$×3CH$_3$CN**).

Quantum chemical calculations were performed using the quantum computing suite ORCA 5.0.4.[12,13] Geometry optimization was performed using (un)restricted Kohn-Sham orbitals density functional theory (DFT RKS/UKS) and the B3LYP functional[14,15] in combination with Ahlrichs' split-valence triple-ζ basis set def2-TZVPP for all atoms[16,17] with the auxiliary basis set SARC/J.[18] Tight convergence criteria were chosen for all calculations (keywords *tightscf* and *tightopt*). All DFT calculations make use of the resolution of identity (Split-RI-J) approach for the Coulomb term in combination with the chain-of-spheres approximation for the exchange term (keyword *RIJCOSX*).[19,20] The zeroth order regular approximation was used to describe relativistic effects in all calculations (keyword *ZORA*).[18] To account for solvent effects, a conductor-like screening model (keyword *CPCM*) was used in all calculations.[21,22] Atom-pairwise dispersion correction was performed with the Becke-Johnson damping scheme (keyword *D3BJ*).[23,24] A numerical frequency calculation confirmed that the optimized geometry corresponds to a minimum structure. Explicit counter ions and/or solvent molecules were not considered. Seventy spin-allowed transitions were calculated by TD-DFT. The charge transfer number analyses of the TD-DFT-calculated transitions were done using *TheoDORE* 2.4 with dividing the complex into three fragments vanadium, ligand #1 and ligand #2.[25]

As DFT and TD-DFT calculations cannot properly resolve the multiplet structure of orbitally degenerate states,[26] and DFT calculated energies of spin-flip states are often at odds with the experimental values,[27-29] we employed CASSCF-SC-NEVPT2 methods in addition. However, the charge transfer character of the excited states is neglected due to the limited active space.

CASSCF(7,12)-SC-NEVPT2 calculations of ground and excited state properties with respect to pure metal-centered (MC) states were performed using the complete-active-space self-consistent field method including spin-orbit coupling (SOC-CASSCF) in conjunction with the fully internally contracted N-electron valence perturbation theory to second order (SC-NEVPT2) in order to recover missing dynamic electron correlation. All electronic states are classified by irreducible representations of the O point group, in spite of the lower actual symmetry of the complex. To model the ligand field accurately, the active space was expanded to encompass the dominant bonding/antibonding orbitals formed between vanadium and the ligands. In addition to the minimal active space of (3,5), two occupied V–N σ bonding orbitals and a second d shell were included in these calculations giving an active space of (7,12). 10 quartet and 10 doublet roots were calculated.

Synthesis of [V(tpe)$_2$][BPh$_4$]$_2$: [V(NCCH$_3$)$_6$][BPh$_4$]$_2$ [2] (102.40 mg, 109.43 µmol, 1.00 eq) and the ligand tpe[1] (282.08 mg, 1095.17 µmol, 10.01 eq) were suspended in a mixture of acetonitrile and diglyme (1:4 *v*/*v*, 30 mL). The reaction was carried out in the microwave and heated for 2 h at 120 °C (150 W). The dark purple precipitate was collected by filtration, washed with diethyl ether (3 × 10 mL) and methanol (3 × 5 mL). Drying under reduced pressure gave **[V(tpe)$_2$][BPh$_4$]$_2$** as a dark purple solid (95.00 mg, 78.38 µmol, 72 %). Dark purple rectangular crystals of **[V(tpe)$_2$][BPh$_4$]$_2$** (9.60 mg, 7.92 µmol) were collected by diethyl ether diffusion in a concentrated acetonitrile solution saturated with [^{n}Bu$_4$N][PF$_6$] (no anion exchange occurs under these conditions, but the higher ionic strength facilitates crystallization). Crystals of **[V(tpe)$_2$][BPh$_4$]$_2$×2DMF** suitable for XRD formed by diethyl ether diffusion in a concentrated solution of **[V(tpe)$_2$][BPh$_4$]$_2$** (9.4 mg, 7.8 mmol) in DMF (0.5 mL) saturated with [^{n}Bu$_4$N][BF$_4$] after two weeks. Elemental analysis for $C_{82}H_{72}B_2N_6OV$: calcd. C 80.07 %, H 5.90 %, N 6.83 %; found C 80.32 %, H 6.25 %, N 6.81 %. ESI$^+$ HRMS (CH$_3$CN): calcd. for $[C_{34}H_{30}N_6V]^{2+}$ *m/z* = 286.5981; found: *m/z* = 286.5971 (100 %); calcd. for $[C_{58}H_{50}BN_6V]^+$ = 892.3625; found: *m/z* = 892.3641 (37 %). CV (CH$_3$CN/[^{n}Bu$_4$N][PF$_6$]): $E_{1/2}$ = –0.05 V (rev.), –2.08 (qrev.) versus FcH$^+$/FcH. IR (ATR): $\tilde{\nu}$ / cm^{-1} = 3053 (w), 2980 (w), 1592 (w), 1580 (w), 1480 (w), 1461 (m), 1437 (w), 1422 (w), 1390 (w), 1296 (w), 1263 (w), 1167 (w), 1137 (w), 1055 (w), 1031 (w), 841 (w), 767 (m), 757 (m), 732 (s), 700 (s), 637 (m), 612 (s), 563 (w), 506 (w), 484 (w), 467 (w), 441 (m). Raman: $\tilde{\nu}$ / cm^{-1} = 3116 (w), 3069 (w), 3029 (w), 1593 (m), 1580 (m), 1475 (w), 1428 (w), 1310 (w), 1309 (w), 1293 (w), 1199 (w), 1167 (w), 1149 (w), 1106 (w), 1059 (w), 1025 (s), 997 (m), 848 (w), 767 (w), 780 (w), 719 (m), 668 (w), 649 (m), 633 (m), 620 (w), 562 (w), 506 (w), 430 (w); broad band between 3500 – 1000.

Synthesis of [V(tpe)$_2$]Cl$_2$: [V(tpe)$_2$][BPh$_4$]$_2$ (15.00 mg, 23.27 µmol) was dissolved in acetone (120 mL) and treated with a saturated solution of [^{n}Bu$_4$N]Cl in acetone (1 mL). Dark purple needles of **[V(tpe)$_2$]Cl$_2$** formed overnight. Elemental analysis for $C_{34}H_{46}Cl_2N_6O_8V$: calcd. C 51.78 %, H 5.88 %, N 10.66 %; found C 51.46 %, H 6.01%, N 10.34 %. ESI$^+$ HRMS (CH$_3$CN): calcd. for $[C_{34}H_{30}N_6V]^{2+}$ *m/z* = 286.5981; found: *m/z* = 286.5971 (100 %); calcd. for $[C_{34}H_{30}ClN_6V]^+$ = 608.1655; found: *m/z* = 608.1644 (59 %). CV (CH$_3$CN/[^{n}Bu$_4$N][PF$_6$]): $E_{1/2}$ = –0.06 V (rev.), –2.08 (qrev.) versus FcH$^+$/FcH. IR (ATR): $\tilde{\nu}$ / cm^{-1} = 3400 (s, b), 3357 (s, b), 3212 (b), 3108 (w), 3075 (w), 2902 (w), 1698 (w), 1641 (m), 1594 (s), 1567 (m), 1461 (s), 1435 (s), 1384 (m), 1310 (w), 1292 (w), 1165 (m), 1145 (w), 1110 (w), 1090 (w), 1071 (w), 1057 (w), 1025 (m), 992 (w), 967 (w), 931 (w), 859 (w), 849 (w), 788 (w), 755 (s), 639 (m), 527 (m), 506 (s, b). Raman: $\tilde{\nu}$ / cm^{-1} = 3081 (w), 3051 (w), 2903 (w), 2871 (w), 1946 (w), 1666 (w), 1595 (m), 1562 (w), 1475 (w), 1437 (w), 1311 (w), 1293 (w), 1200 (w), 1149 (w), 1111 (w), 1058 (w), 1020 (s), 854 (w), 778 (w), 719 (m), 649 (m), 634 (m), 570 (w), 504 (w); weak broad band between 3500 – 1000. UV/vis/NIR (H$_2$O): λ (ε) = 571 (7835), 442 (3380), 435 (4070), 325 nm (1375 M^{-1} cm^{-1}).

Synthesis of [V(tpe)$_2$][PF$_6$]$_2$: [V(tpe)$_2$]Cl$_2$ (15.00 mg, 23.27 µmol) was dissolved in methanol (4 mL) and treated with a saturated solution of [^{n}Bu$_4$N][PF$_6$] in methanol (1 mL). Dark purple rectangular plates of **[V(tpe)$_2$]Cl$_2$** formed after diffusion of diethyl ether into the methanol solution under ambient conditions. Elemental analysis for $C_{34}H_{30}F_{12}N_6P_2V$: calcd. C 47.29 %, H 3.50 %, N 9.73 %; found C 47.31 %, H 3.87 %, N 9.72 %. ESI$^+$ HRMS (CH$_3$CN): calcd. for $[C_{34}H_{30}N_6V]^{2+}$ *m/z* = 286.5981; found: *m/z* = 286.5971 (100 %); calcd. for $[C_{34}H_{30}F_6N_6PV]^+$ = 718.1608; found: *m/z* = 718.1595 (87 %). CV (CH$_3$CN/[^{n}Bu$_4$N][PF$_6$]): $E_{1/2}$ = –0.06 V (rev.), –2.08 (qrev.) versus FcH$^+$/FcH. IR (ATR): $\tilde{\nu}$ / cm^{-1} = 3082 (w), 1596 (w), 1463 (m), 1437 (w), 1390 (w), 1294 (w), 1174 (w), 1104 (w), 1053 (w), 1023 (w), 831 (s, PF$_6$), 778 (m), 749 (m), 635 (m), 555 (s, PF$_6$), 512 (w), 498 (w). Raman: $\tilde{\nu}$ / cm^{-1} = 3140 (w), 3083 (w), 1594 (m), 1476 (w), 1429 (w), 1311 (w), 1294 (w), 1199 (w), 1169 (w), 1145 (w), 1106 (w), 1059 (w), 1022 (s), 850 (w), 774 (w), 740 (w, PF$_6$), 718 (m), 649 (m), 634 (w), 563 (w), 511 (w); broad band between 3500 – 1000.

Synthesis of [V(tpe)$_2$][BF$_4$]$_2$: [V(tpe)$_2$]Cl$_2$ (15.00 mg, 23.27 µmol) was dissolved in methanol (4 mL) and excess of NaBF$_4$ was added as a solid. Dark purple crystals of **[V(tpe)$_2$][BF$_4$]$_2$** formed within 14 days under ambient conditions. ESI$^+$ HRMS (CH$_3$CN): calcd. for $[C_{34}H_{30}N_6V]^{2+}$ *m/z* = 286.5981; found: *m/z* = 286.5971 (99 %); calcd. for $[C_{34}H_{30}BF_4N_6V]^+$ = 660.1996; found: *m/z* = 660.1996 (100 %). IR (ATR): $\tilde{\nu}$ / cm^{-1} = 3641 (w), 3551 (w), 3092 (w), 1622 (w), 1594 (m), 1463 (m), 1437 (w), 1390 (w), 1282 (w), 1294 (w), 1172 (w), 1104 (w), 1053 (s, BF$_4$), 1023 (s), 1000 (s), 845 (w), 774 (m), 755 (m), 636 (m), 561 (w), 518 (m), 441 (m). Raman: $\tilde{\nu}$ / cm^{-1} = 3093 (w), 1593 (m), 1475 (w), 1430 (w), 1309 (w), 1294 (w), 1200 (w), 1169 (w), 1147 (w), 1110 (w), 1059 (w), 1022 (s), 853 (w), 770 (w), 718 (m), 649 (m), 634 (w), 562 (w), 512 (w); broad band between 3500 – 1000.

Crystallographic Data of [V(tpe)$_2$][BPh$_4$]$_2$. $C_{82}H_{70}B_2N_6V$ (1212.00); triclinic; $P\bar{1}$, a = 10.536(2) Å, b = 12.241(3) Å, c = 13.872(3) Å, α = 96.90(3)°, β = 109.46(3)°, γ = 106.76(3)°; V = 1568.3(7) Å^3, Z = 1; density (calculated) = 1.283 g cm^{-3}; T = 120(2) K; μ = 0.212 mm^{-1}; F(000) = 637; crystal size 0.630×0.473×0.360 mm^3; θ = 2.674 to 28.138 deg.; –13≤h≤13, –16≤k≤16, –17≤l≤18; rfln collected = 13788; rfln unique = 7505 [R(int) = 0.0261]; completeness to θ = 25.242 deg. = 99.4 %; semi empirical absorption correction from integration; max. and min. transmission 0.9402 and 0.8880; data 7505; restraints 0; parameters 413; goodness-of-fit on F^2 = 1.034; final indices [$I > 2\sigma(I)$] R_1 = 0.0396, wR_2 = 0.0954; R indices (all data) R_1 = 0.0452, wR_2 = 0.1000; largest diff. peak and hole 0.415 and –0.572 e Å^{-3}.

Crystallographic Data of [V(tpe)$_2$][BPh$_4$]$_2$×2DMF. $C_{88}H_{84}B_2N_8O_2V$ (1358.19); monoclinic; $P2_1$, a = 12.620(3) Å, b = 12.347(3) Å, c = 22.865(5) Å, α = 90°, β = 100.16(3)°, γ = 90°; V = 3506.9(13) Å^3, Z = 2; density (calculated) = 1.286 g cm^{-3}; T = 120(2) K; μ = 0.200 mm^{-1}; F(000) = 1434; crystal size 0.910×0.513×0.310 mm^3; θ = 2.449 to 28.089 deg.; –16≤h≤16, –16≤k≤16, –30≤l≤30; rfln collected = 34675; rfln unique = 16910 [R(int) = 0.0278]; completeness to θ = 25.242 deg. = 99.8 %; semi empirical absorption correction from integration; max. and min. transmission 0.9402 and 0.8880; data 16910; restraints 177; parameters 965; goodness-of-fit on F^2 = 1.055; final indices [$I > 2\sigma(I)$] R_1 = 0.0447, wR_2 = 0.1049; R indices (all data) R_1 = 0.0544, wR_2 = 0.1111; largest diff. peak and hole 0.417 and –0.217 e Å^{-3}.

Crystallographic Data of [V(tpe)$_2$]Cl$_2$×2H$_2$O×acetone. $C_{37}H_{40}Cl_2N_6O_3V$ (738.59); monoclinic; $P2_1$/c, a = 9.7284(19) Å, b = 23.142(5) Å, c = 15.824(3) Å, α = 90°, β = 100.50(3)°, γ = 90°; V = 3502.9(13) Å^3, Z = 4; density (calculated) = 1.401 g cm^{-3}; T = 120(2) K; μ = 0.482 mm^{-1}; F(000) = 1540; crystal size 0.700×0.260×0.030 mm^3; θ = 2.451 to 28.026 deg.; –12≤h≤12, –30≤k≤30, –20≤l≤20; rfln collected = 30784; rfln unique = 8351 [R(int) = 0.3395]; completeness to θ = 25.242 deg. = 99.9 %; semi empirical absorption correction from integration; max. and min. transmission 0.9896 and 0.8118; data 8351; restraints 0; parameters 455; goodness-of-fit on F^2 = 1.118; final indices [$I > 2\sigma(I)$] R_1 = 0.1141, wR_2 = 0.2365; R indices (all data) R_1 = 0.2208, wR_2 = 0.3067; largest diff. peak and hole 0.763 and –0.693 e Å^{-3}.

Crystallographic Data of [V(tpe)$_2$][PF$_6$]$_2$×0.5MeOH. $C_{34.50}H_{32}F_{12}N_6O_{0.5}P_2V$ (879.54); triclinic; $P\bar{1}$, a = 11.858(2) Å, b = 13.279(3) Å, c = 15.402(3) Å, α = 74.69(3)°, β = 67.56(3)°, γ = 64.09(3)°; V = 2002.6(10) Å^3, Z = 2; density (calculated) = 1.459 g cm^{-3}; T = 120(2) K; μ = 0.418 mm^{-1}; F(000) = 892; crystal size 0.180×0.130×0.040 mm^3; θ = 2.367 to 28.026 deg.; –15≤h≤15, –17≤k≤17, –20≤l≤20; rfln collected = 38471; rfln unique = 9582 [R(int) = 0.0978]; completeness to θ = 25.242 deg. = 99.9 %; semi empirical absorption correction from equivalents; max. and min. transmission 1.17178 and 0.90689; data 9582; restraints 1885; parameters 862; goodness-of-fit on F^2 = 1.062; final indices [$I > 2\sigma(I)$] R_1 = 0.0986, wR_2 = 0.2705; R indices (all data) R_1 = 0.1468, wR_2 = 0.3101; largest diff. peak and hole 1.440 and –0.735 e Å^{-3}.

Crystallographic Data of [V(tpe)$_2$][BF$_4$]$_2$×MeOH. $C_{35}H_{34}F_8N_6OB_2V$ (779.24); triclinic; $P\bar{1}$, a = 11.063(2) Å, b = 12.812(3) Å, c = 13.686(3) Å, α = 87.51(3)°, β = 72.16(3)°, γ = 66.86(3)°; V = 1691.4(8) Å^3, Z = 2; density (calculated) = 1.530 g cm^{-3}; T = 120(2) K; μ = 0.378 mm^{-1}; F(000) = 798; crystal size 0.640×0.360×0.130 mm^3; θ = 2.934 to 27.926 deg.; –14≤h≤14, –16≤k≤16, –17≤l≤17; rfln collected = 17359; rfln unique = 8050 [R(int) = 0.0329]; completeness to θ = 25.242 deg. = 99.8 %; semi empirical absorption correction from equivalents; max. and min. transmission 1.15155 and 0.91592; data 8050; restraints 208; parameters 532; goodness-of-fit on F^2 = 1.050; final indices [$I > 2\sigma(I)$] R_1 = 0.0395, wR_2 = 0.1033; R indices (all data) R_1 = 0.0451, wR_2 = 0.1077; largest diff. peak and hole 0.705 and –0.348 e Å^{-3}.

Crystallographic Data of [VO(κ^2-tpe)(tpe)][PF$_6$]$_2$×3CH$_3$CN. $C_{40}H_{39}F_{12}N_9OP_2V$ (1002.68); monoclinic; $P2_1$, a = 11.660(2) Å, b = 13.160(3) Å, c = 14.158(3) Å, α = 90°, β = 95.93(3)°, γ = 90°; V = 2160.9(8) Å^3, Z = 2; density (calculated) = 1.541 g cm^{-3}; T = 120(2) K; μ = 0.401 mm^{-1}; F(000) = 1022; crystal size 0.510×0.263×0.250 mm^3; θ = 2.341 to 28.085 deg.; –14≤h≤15, –17≤k≤17, –18≤l≤18; rfln collected = 24221; rfln unique = 10441 [R(int) = 0.0540]; completeness to θ = 25.242 deg. = 99.8 %; semi empirical absorption correction from equivalents; max. and min. transmission 1.0944 and 0.9115; data 10441; restraints 385; parameters 656; goodness-of-fit on F^2 = 1.063; final indices [$I > 2\sigma(I)$] R_1 = 0.0543, wR_2 = 0.1309; R indices (all data) R_1 = 0.0678, wR_2 = 0.1403; largest diff. peak and hole 1.001 and –0.529 e Å^{-3}; Flack-X 0.028(13).

Photocatalysis experiments were conducted using 560 nm or 625 nm UHP LED lamps (*Prizmatix*) as excitation sources. The collimated beam was focused inside the cuvette with a plano convex lens. The beam diameter was adjusted to 0.5 cm at the sample position. The temperature of the irradiated solution was kept at 278 K or 293 K with a Peltier module. Air was continuously bubbled into the solution with a pump to keep the concentration of oxygen in the solution constant. ^{1}H NMR spectra were recorded at the indicated times (Fig. S18 and S20).

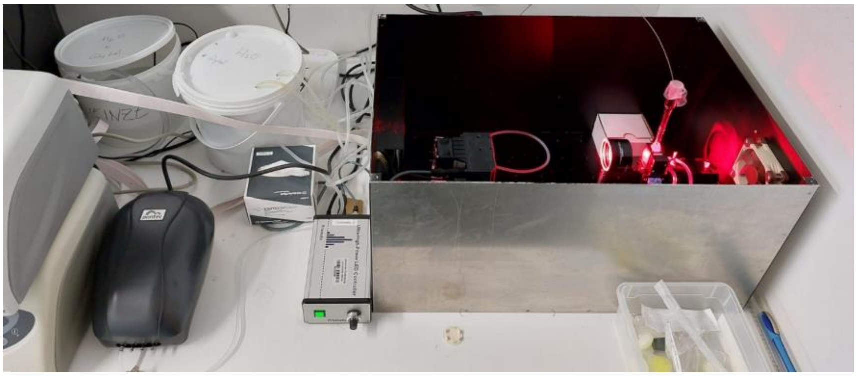

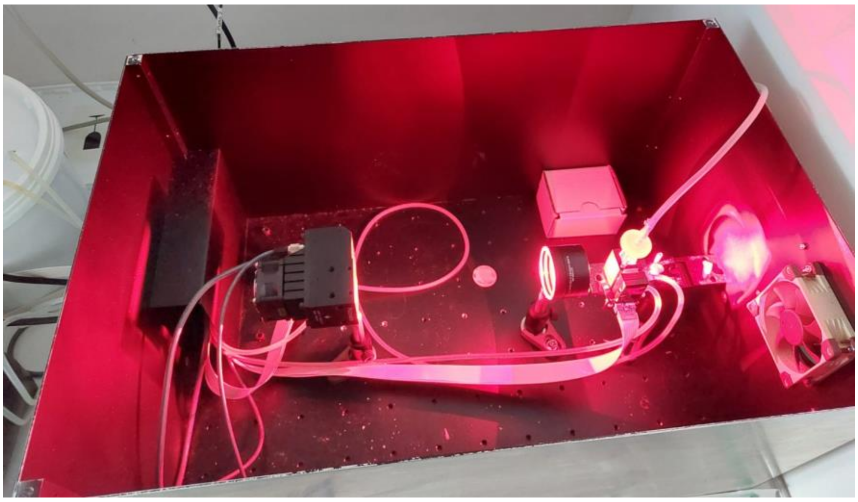

<u>5-HMF oxidation:</u>

^{1}H NMR (5-HMF, D_2O/buffer): δ / ppm = 9.43 (s, 1H), 7.51 (d, $^{3}J_{HH}$ = 3.7 Hz, 1H), 6.65 (d, $^{3}J_{HH}$ = 3.7 Hz, 1H), 4.67 (s, 2H).

^{1}H NMR ((*Z*)-5-hydroxy-4-keto-2-pentenoate, D_2O/buffer): δ / ppm = 6.47 (d, $^{3}J_{HH}$ = 12.0 Hz, 1H), 6.22 (d, $^{3}J_{HH}$ = 12.0 Hz, 1H), 4.41 (s, 2H).

^{1}H NMR (formiate, D_2O/buffer): δ / ppm = 8.43 (s).

<u>1-MCH oxidation:</u>

^{1}H NMR (1-MCH, CD_3CN): δ / ppm = 5.38 (m, 1H), 1.64–1.49 (m, 7H); other resonances are masked by the solvent; the resonance at 5.38 ppm is monitored.[32]

^{1}H NMR (2-methyl-cyclohex-2-enyl hydroperoxide, CD_3CN): δ / ppm = 9.35 (s, 1H), 5.68 (br s, 1H), 4.17 (br s, 1H); other resonances are masked by the solvent; the resonance at 5.68 ppm is monitored.[33]

^{1}H NMR (3-hydroperoxy-3-methylcyclohex-1-ene, CD_3CN): δ / ppm = 8.76 (s, 1H), 5.88 (m, 1H), 5.53 (m, 1 H), 1.23 (s, 3H); other resonances are masked by the solvent; the resonance at 5.88 ppm is monitored.[33]

^{1}H NMR (1-hydroperoxy-2-methylenecyclohexane, CD_3CN): δ / ppm = 9.28 (s, 1H), 4.84 (d, $^{3}J_{HH}$ = 4.7 Hz, 2H), 4.29 (t, $^{3}J_{HH}$ = 4.7 Hz, 1H); other resonances are masked by the solvent; the resonance at 4.84 ppm is monitored.[33]

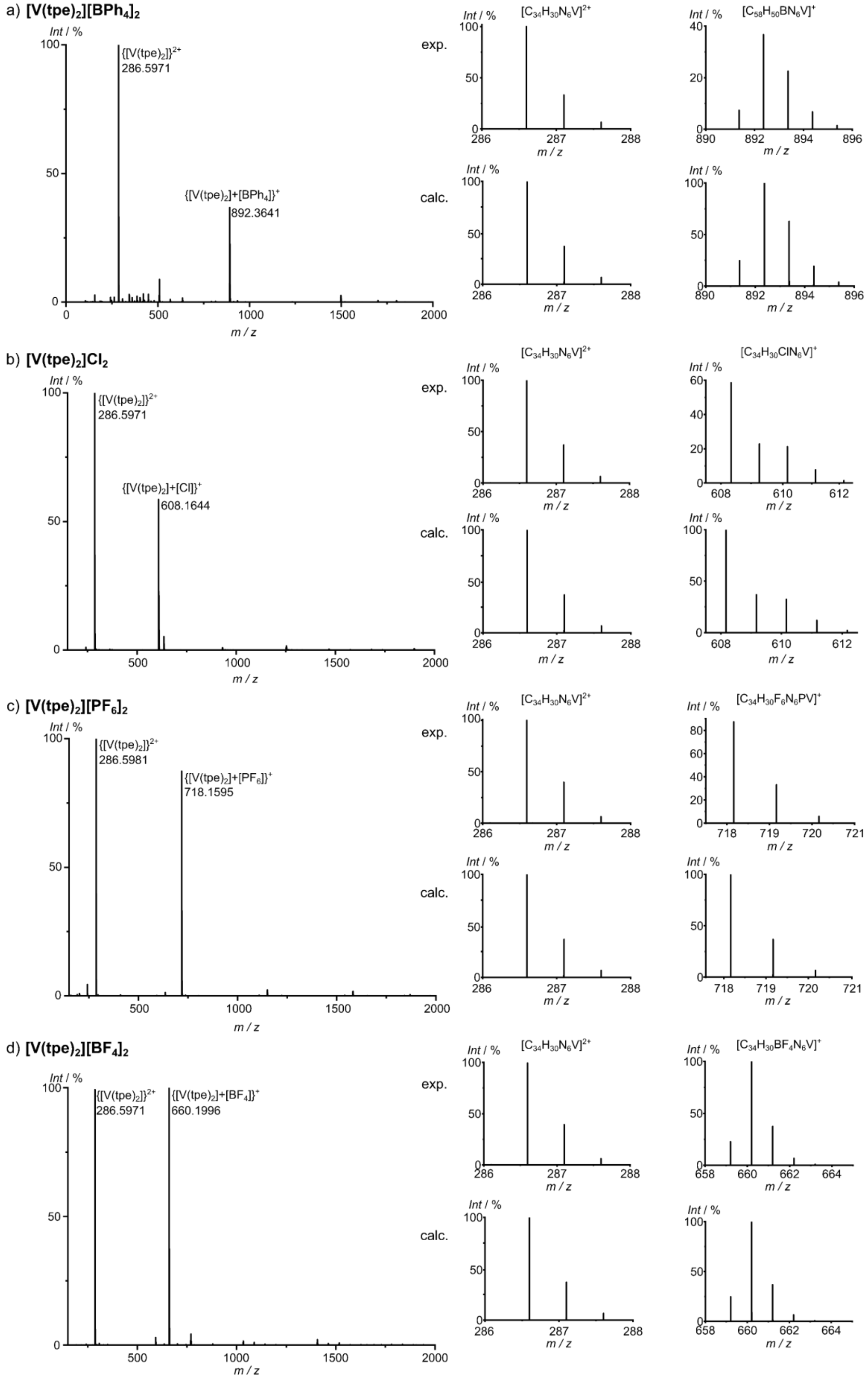

Figure S1. ESI$^+$ mass spectra of a) **[V(tpe)$_2$][BPh$_4$]$_2$**, b) **[V(tpe)$_2$]Cl$_2$**, c) **[V(tpe)$_2$][PF$_6$]$_2$** and c) **[V(tpe)$_2$][BF$_4$]$_2$** in CH_3CN. The insets show the calculated and experimental isotope distribution of the dication **[V(tpe)$_2$]$^{2+}$** and the ion cluster **{[V(tpe)$_2$][X]}$^+$** (X = BPh_4, Cl, PF_6, BF_4), respectively.

Table S1. Bond lengths [Å] and angles [deg] of **[V(tpe)$_2$][BPh$_4$]$_2$**, **[V(tpe)$_2$][BPh$_4$]$_2$×2DMF**, **[V(tpe)$_2$]Cl$_2$×2 H$_2$O×acetone**, **[V(tpe)$_2$][BF$_4$]$_2$×MeOH** (two independent centrosymmetric cations in the unit cell) and **[V(tpe)$_2$][PF$_6$]$_2$×0.5MeOH** (two independent centrosymmetric cations in the unit cell) from single crystal XRD measurements.

bond lengths	**[V(tpe)$_2$][BPh$_4$]$_2$**	**[V(tpe)$_2$][BPh$_4$]$_2$ ×2DMF**	**[V(tpe)$_2$]Cl$_2$ ×2H$_2$O ×acetone**	**[V(tpe)$_2$][BF$_4$]$_2$ ×MeOH**	**[V(tpe)$_2$][PF$_6$]$_2$ ×0.5MeOH**
V1–N1	2.0978(15)	2.120(3)	2.095(6)	2.1021(15)	2.106(4)
V1–N2	2.1164(12)	2.110(2)	2.116(6)	2.0984(14)	2.122(4)
V1–N3	2.1051(13)	2.105(3)	2.100(6)	2.1134(15)	2.119(4)
V1–N4		2.108(3)	2.121(6)		
V1–N5		2.104(2)	2.117(6)		
V1–N6		2.110(3)	2.109(6)		
V2–N4				2.1176(14)	2.125(5)
V2–N5				2.1065(14)	2.116(5)
V2–N6				2.1170(14)	2.117(4)
**V–N$_{average}$**	**2.1064**	**2.110**	**2.110**	**2.1092**	**2.118**
angles					
N1–V1–N2	85.33(6)	84.25(10)	85.8(2)	84.73(6)/84.73(6)	83.75(17)
N1–V1–N3	84.80(5)	85.29(11)	84.8(2)	85.13(6)/85.13(6)	84.39(16)
N2–V1–N3	85.19(5)	85.42(10)	84.7(2)	85.71(6)/85.71(6)	84.31(17)
N5–V1–N4		84.89(10)	85.1(2)		
N6–V1–N4		84.94(10)	84.2(2)		
N6–V1–N5		84.79(11)	85.1(2)		
N5–V2–N4				84.66(6)/84.66(6)	84.86(18)
N6–V2–N4				84.28(6)/84.28(6)	84.06(18)
N6–V2–N5				84.44(6)/84.44(6)	83.68(18)
**N–V–N$_{average}$**	**85.11**	**84.90**	**84.92**	**84.83**	**84.18**
N1–V1–N1‘	180.00	178.56(12)	179.7(3)	180.00	180.00
N2–V1–N2‘	180.00	179.12(11)	178.8(2)	180.00	180.00
N3–V1–N3‘	180.00	178.08(12)	179.0(3)	180.00	180.00
N4–V2–N4‘				180.00	180.00
N5–V2–N5‘				180.00	180.00
N6–V2–N6‘				180.00	180.00
**N–V–N$_{average}$**	**180.00**	**178.59**	**179.2**	**180.00**	**180.00**

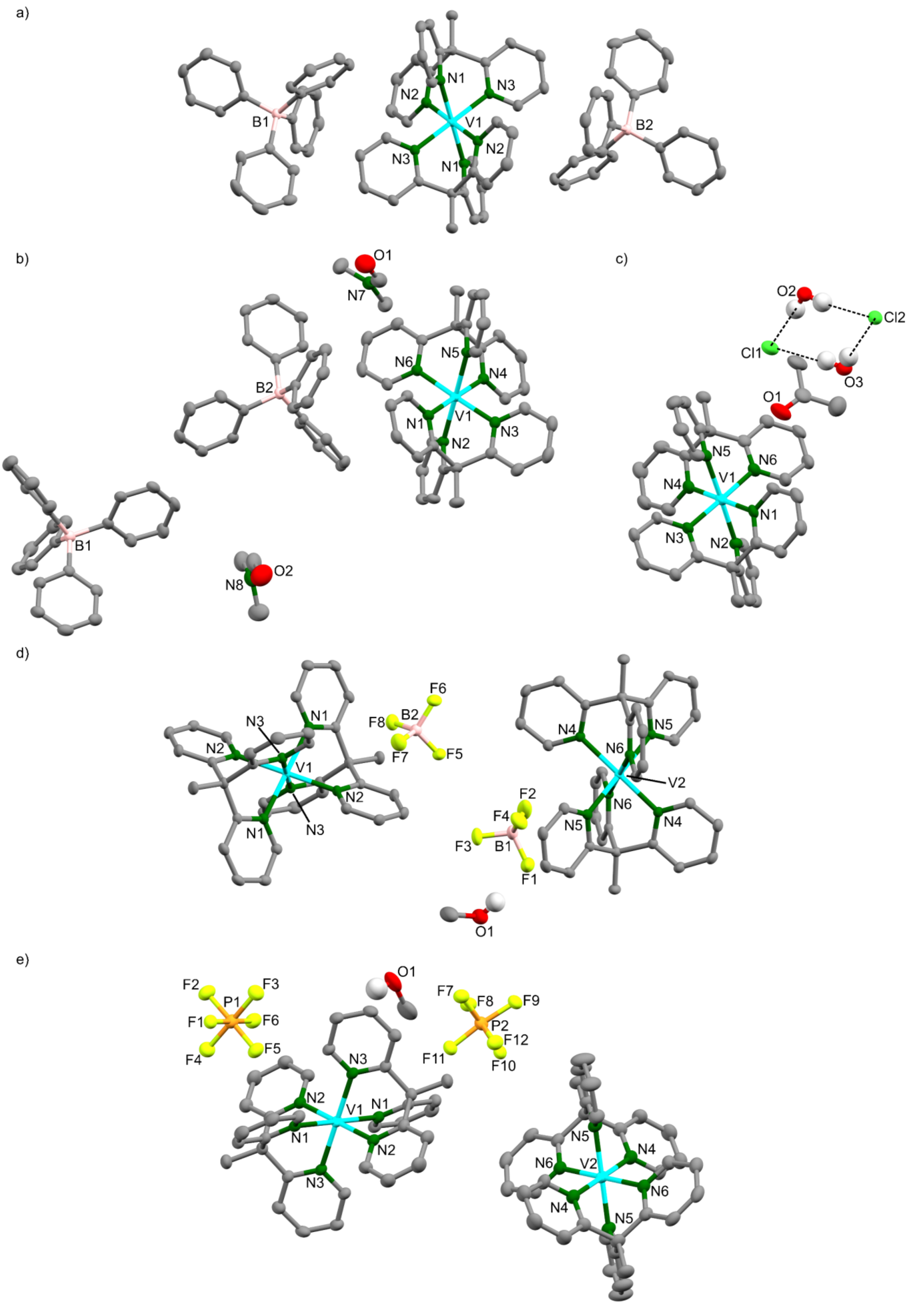

Figure S2. Structures of a) $\mathbf{[V(tpe)_2][BPh_4]_2}$, b) $\mathbf{[V(tpe)_2][BPh_4]_2{\times}2DMF}$, c) $\mathbf{[V(tpe)_2]Cl_2{\times}2H_2O{\times}acetone}$, d) $\mathbf{[V(tpe)_2][BF4_4]_2{\times}MeOH}$ and e) $\mathbf{[V(tpe)_2][PF_6]_2{\times}0.5MeOH}$ with thermal ellipsoids set to 50 % probability. CH hydrogen atoms omitted.

Table S2. Relative energies [eV],[26] bond lengths [Å] and angles [deg] of **[V(tpe)$_2$]$^{2+}$** (quartet ground state, lowest energy doublet state, excited doublet state, lowest energy excited quartet state) obtained from DFT calculations.

	**quartet ground state $^4A_2$**	**lowest energy doublet state**	**excited doublet state, root 1**	**excited quartet state, root 1**
E_{rel}	**0**	**0.65**	**1.10**	**2.07**
V1–N1	2.125	2.103	2.093	2.138
V1–N2	2.125	2.107	2.093	2.100
V1–N3	2.126	2.117	2.094	2.114
V1–N4	2.125	2.103	2.093	2.092
V1–N5	2.124	2.107	2.093	2.102
V1–N6	2.126	2.117	2.094	2.086
**V–N$_{average}$**	**2.125**	**2.109**	**2.093**	**2.105**
N1–V1–N2	84.81	85.17	85.78	85.41
N1–V1–N3	84.80	85.34	85.65	84.81
N2–V1–N3	84.78	85.09	85.64	84.34
N5–V1–N4	84.81	85.34	85.78	84.43
N6–V1–N4	84.80	85.17	85.64	86.99
N6–V1–N5	84.78	85.09	85.64	85.55
**N–V–N$_{average}$**	**84.80**	**85.20**	**85.69**	**85.26**
N1–V1–N1‘	180.00	180.00	180.00	177.66
N2–V1–N2‘	180.00	180.00	180.00	179.79
N3–V1–N3‘	180.00	180.00	180.00	178.94
**N–V–N$_{average}$**	**180.00**	**180.00**	**180.00**	**178.80**

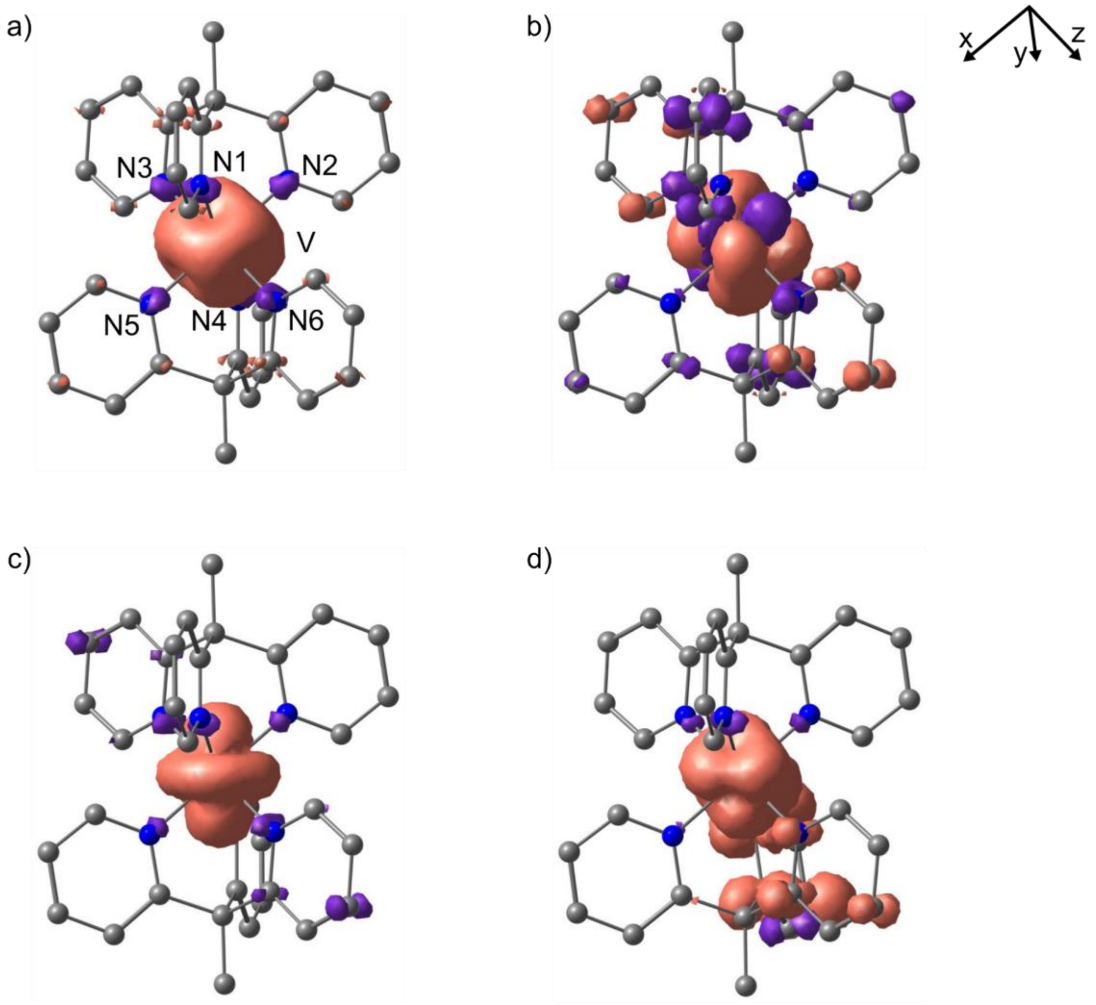

Figure S3. DFT-optimized geometries of **[V(tpe)$_2$]$^{2+}$** in a) the quartet ground state, b) the lowest energy doublet state, c) the excited doublet state and d) the lowest energy excited quartet state obtained from DFT calculations. Hydrogen atoms omitted. α and β spin densities (orange/purple) are displayed with isosurface values of 0.005 and 0.0025 for quartet and doublet states, respectively.

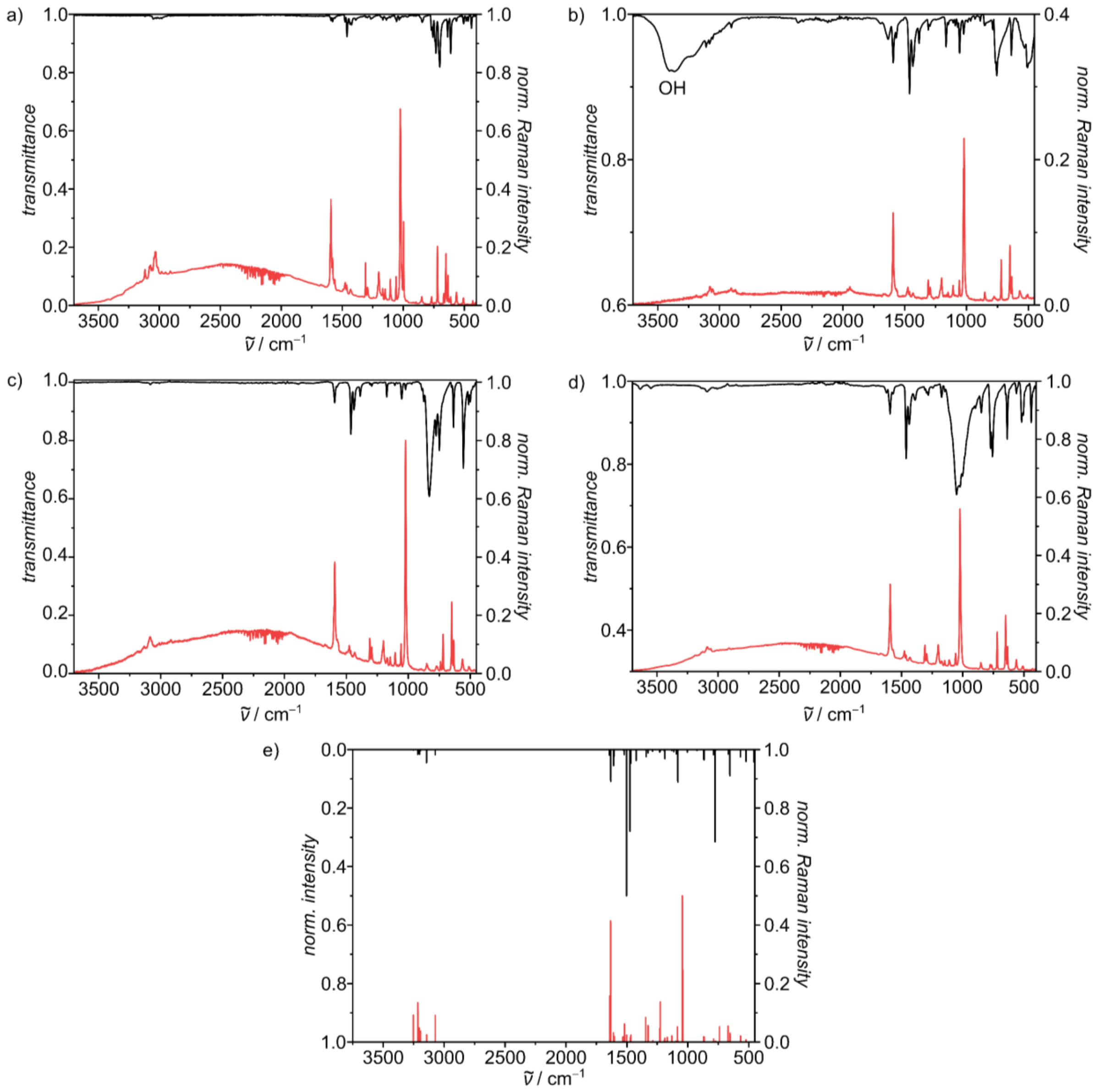

Figure S4. a) ATR-IR (black) and Raman spectra (red) of a) **[V(tpe)$_2$][BPh$_4$]$_2$**, b) **[V(tpe)$_2$]Cl$_2$×2H$_2$O×acetone** and c) **[V(tpe)$_2$][PF$_6$]$_2$** and d) **[V(tpe)$_2$][BF$_4$]$_2$**. e) DFT-calculated IR (black) and Raman spectrum (red) of **[V(tpe)$_2$]**$^{2+}$.

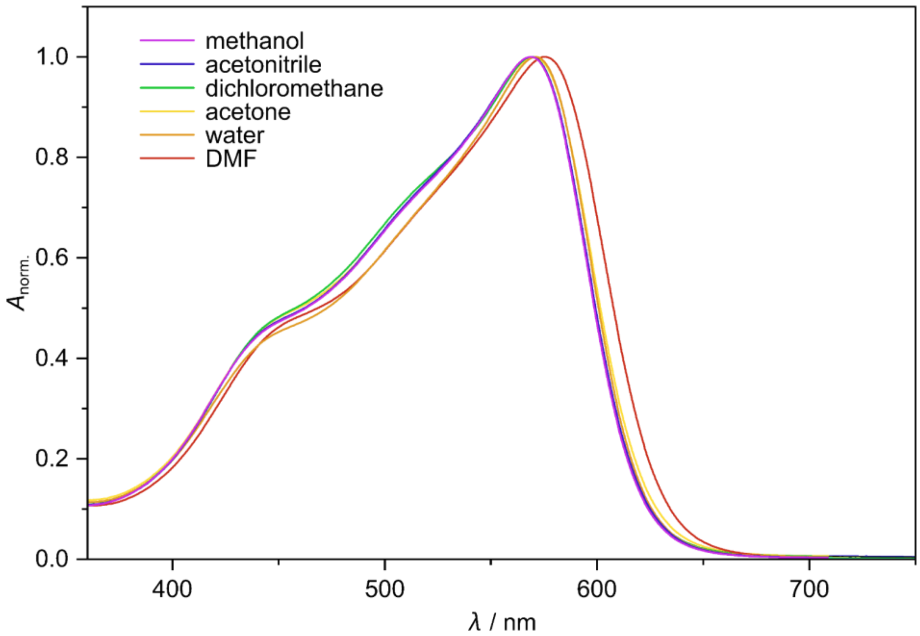

Figure S5. Normalized absorption spectra of **[V(tpe)$_2$]Cl$_2$** in methanol (purple), acetonitrile (blue), dichloromethane (green), acetone (yellow), water (orange) and DMF (red).

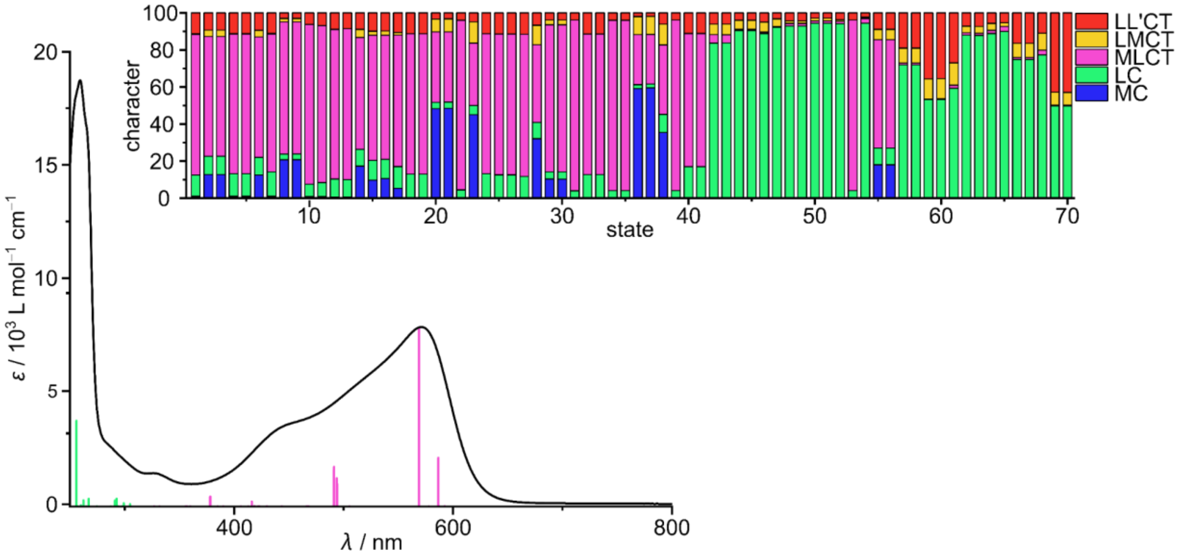

Figure S6. UV/vis absorption spectrum of **[V(tpe)$_2$]Cl$_2$** in water at 293 K (black). TD-DFT-calculated singlet transitions shown as vertical sticks (shifted to lower energy by 923 cm^{-1}). Charge transfer number analysis of **[V(tpe)$_2$]$^{2+}$** from TD-DFT calculations. MC = metal centered (blue), MLCT = metal to ligand charge transfer (pink), LMCT = ligand to metal charge transfer (yellow), LC = ligand centered (green), LL'CT = ligand to ligand charge transfer (red).

Table S3. Seventy TD-DFT-calculated spin-allowed electronic transitions of **$[V(tpe)_2]^{2+}$** with electron difference densities. Hydrogen atoms were omitted. CPCM(acetonitrile) ZORA SARC/J RIJCOSX B3LYP D3BJ ZORA-Def2-TZVPP), orange: gain electron density, purple: loss electron density.

TDDFT difference density (isosurface value 0.001 a.u.)	λ / nm (unshifted)	λ / nm (shifted by 923 cm^{-1} to lower energy)	oscillator strength f	transition number
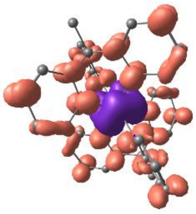	561.7	592.4	2.87E-04	1
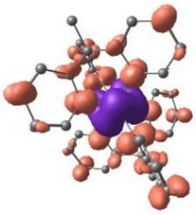	557.4	587.6	9.21E-07	2
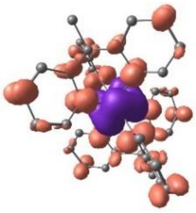	557.3	587.5	1.66E-03	3
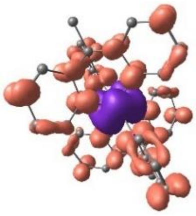	556.6	586.7	5.59E-02	4
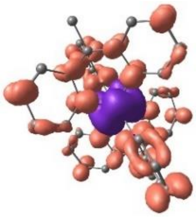	556.6	586.7	5.57E-02	5
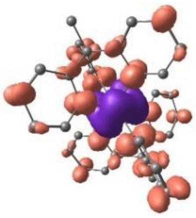	548.7	578.0	5.30E-08	6
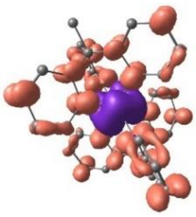	540.6	569.0	2.04E-01	7

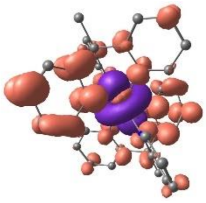	482.8	505.3	0	8
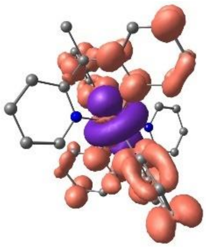	482.6	505.1	1.00E-09	9
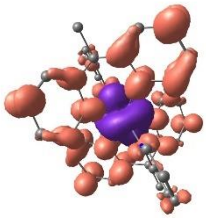	472.6	494.2	2.58E-02	10
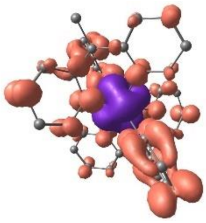	472.2	493.7	3.24E-02	11
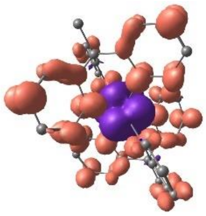	470.0	491.3	4.55E-02	12
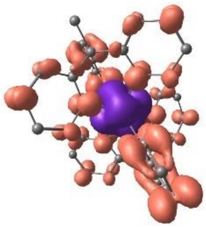	469.8	491.1	4.14E-02	13
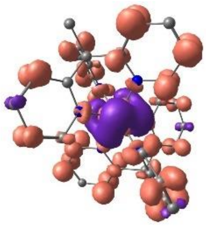	448.7	468.1	1.00E-09	14
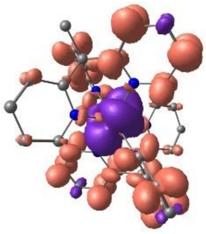	447.6	466.9	0	15

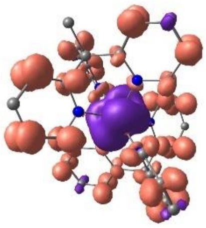	447.5	466.8	1.00E-09	16
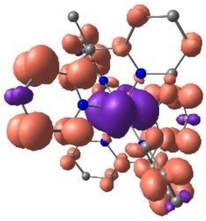	446.9	466.1	0	17
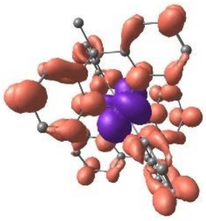	426.1	443.5	0	18
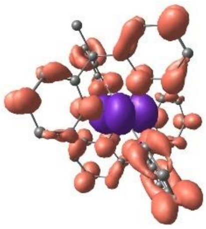	426.0	443.4	0	19
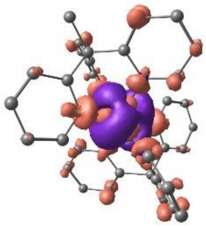	413.5	429.9	0	20
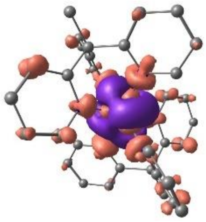	413.0	429.4	1.00E-09	21
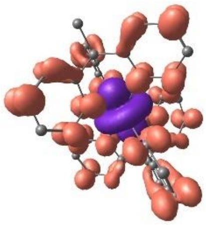	407.0	422.9	5.90E-08	22
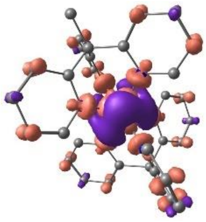	405.8	421.6	2.00E-09	23

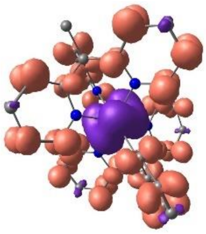	403.0	418.6	2.95E-04	24
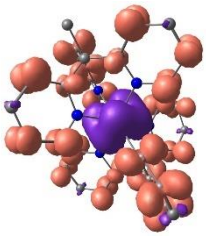	400.7	416.1	5.38E-03	25
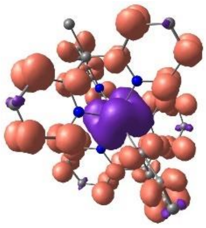	400.7	416.1	5.30E-03	26
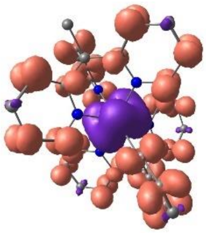	397.6	412.7	6.81E-06	27
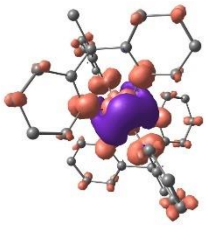	391.5	406.2	0	28
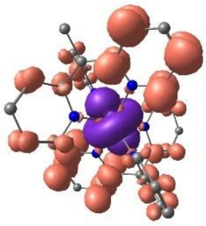	376.6	390.2	0	29
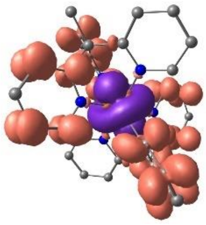	376.4	389.9	0	30

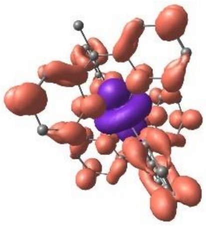	371.6	384.8	0	31
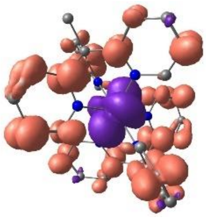	365.5	378.3	1.10E-02	32
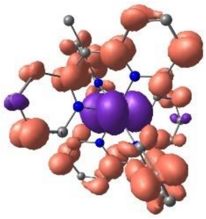	365.3	378.0	1.09E-02	33
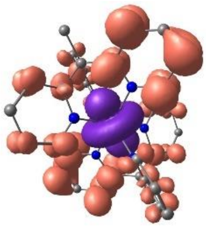	348.3	359.9	3.27E-03	34
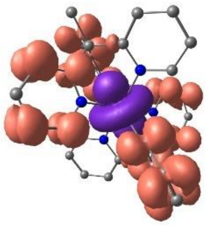	348.2	359.8	5.41E-03	35
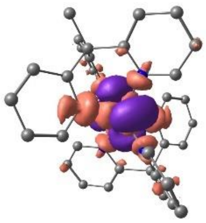	345.4	356.8	0	36
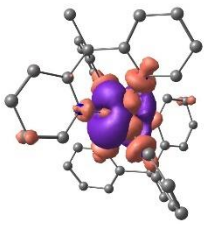	345.2	356.6	0	37
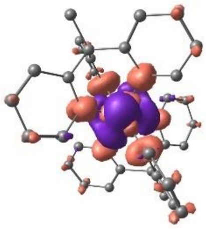	344.2	355.5	0	38

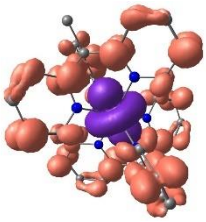	321.9	331.8	2.68E-07	39
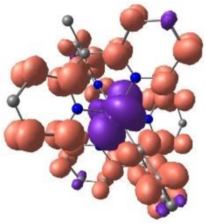	317.4	327.0	0	40
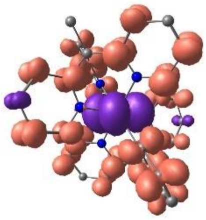	317.3	326.9	0	41
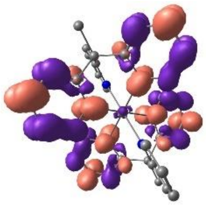	297.0	305.4	5.00E-09	42
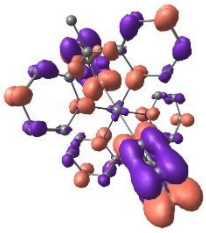	296.9	305.3	2.00E-09	43
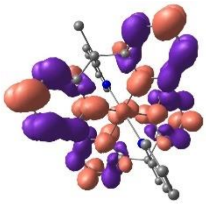	296.5	304.8	2.52E-03	44
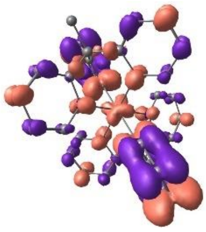	296.4	304.7	2.55E-03	45
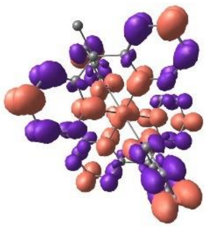	291.1	299.1	3.58E-03	46

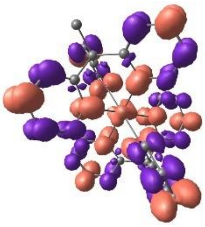	289.7	297.7	0	47
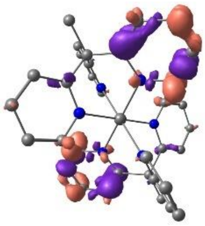	286.5	294.3	1.00E-09	48
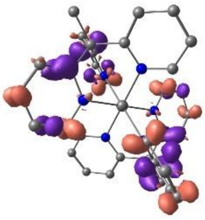	286.5	294.3	1.00E-09	49
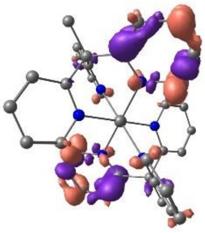	284.9	292.6	8.69E-03	50
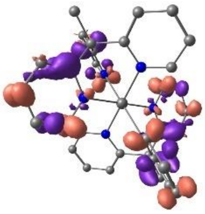	284.8	292.5	8.68E-03	51
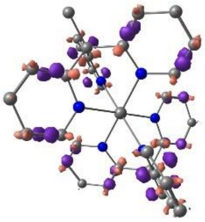	283.3	290.9	6.56E-03	52
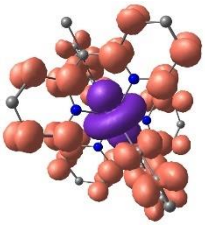	282.8	290.4	0	53
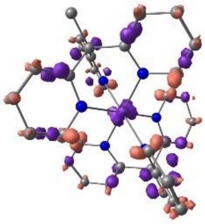	279.8	287.2	0	54

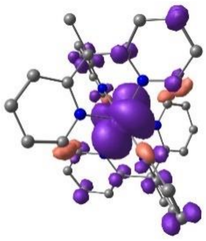	267.7	274.5	0	55
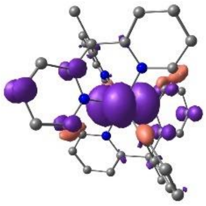	267.7	274.5	0	56
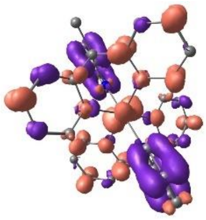	260.7	267.1	8.56E-03	57
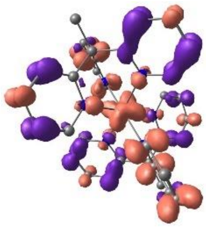	260.6	267.0	8.73E-03	58
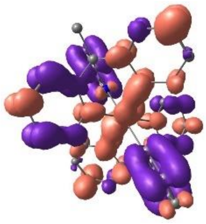	260.3	266.7	1.00E-08	59
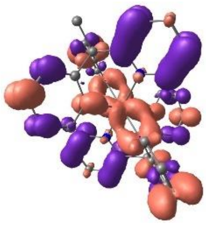	260.2	266.6	4.00E-09	60
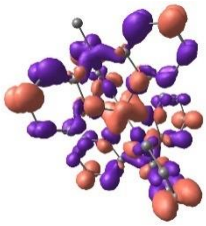	260.1	266.5	2.45E-01	61
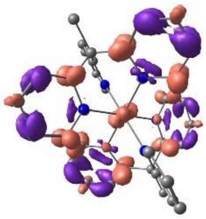	258.3	264.6	1.00E-09	62

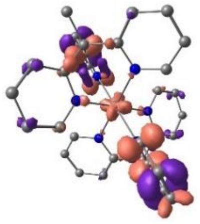	258.3	264.6	1.00E-09	63
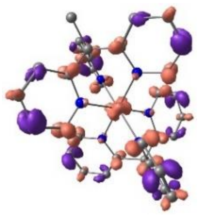	256.0	262.2	6.90E-03	64
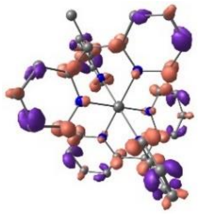	254.2	260.3	0	65
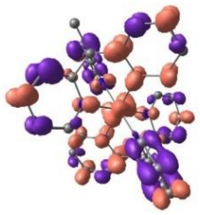	253.8	259.9	1.01E-03	66
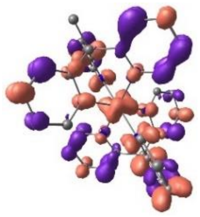	253.7	259.8	1.05E-03	67
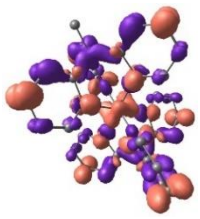	251.1	257.1	4.00E-09	68
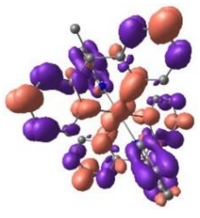	249.9	255.8	9.82E-02	69
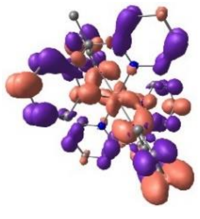	249.8	255.7	9.80E-02	70

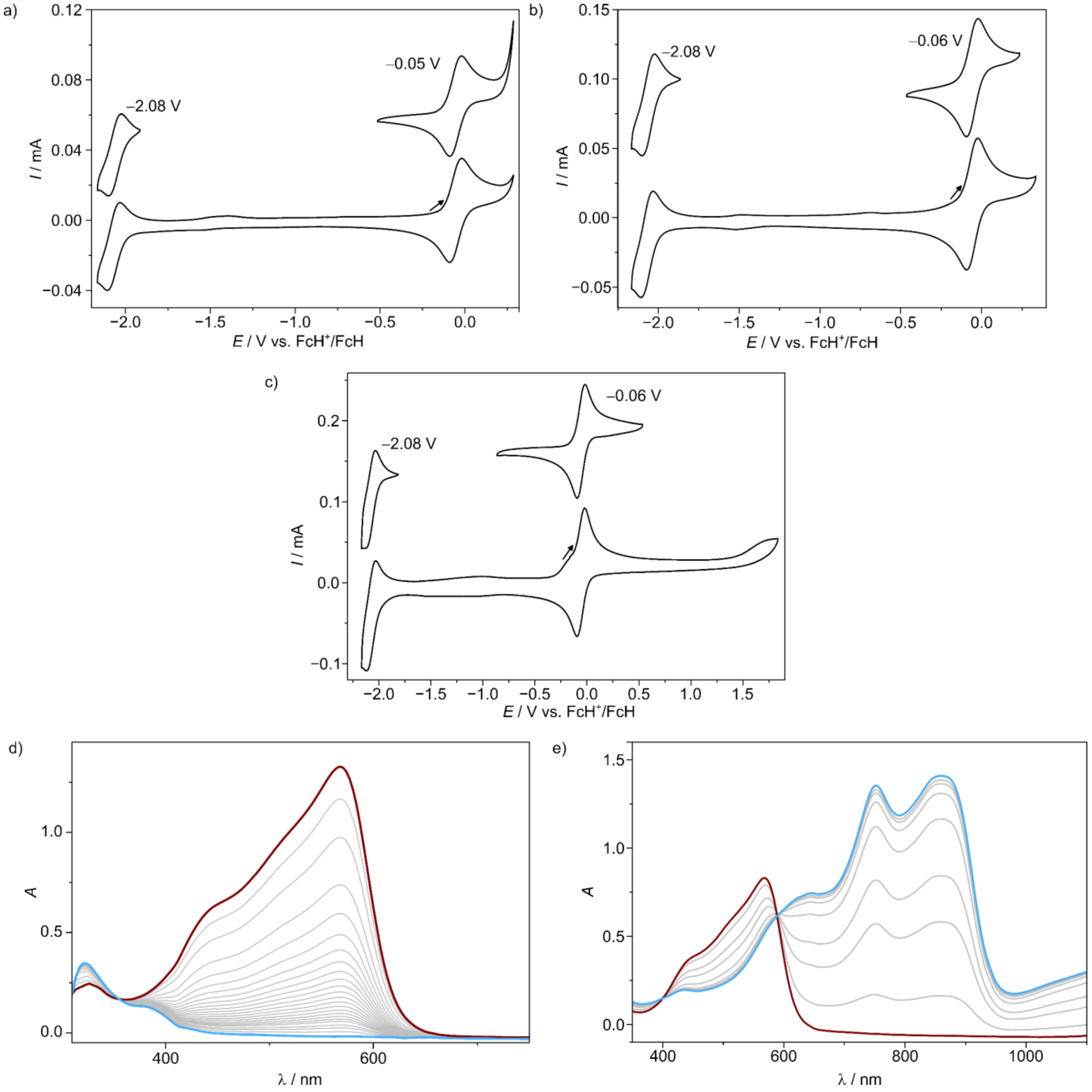

Figure S7. Cyclic voltammograms of a) **[V(tpe)$_2$][BPh$_4$]$_2$**, b) **[V(tpe)$_2$]Cl$_2$** and c) **[V(tpe)$_2$][PF$_6$]$_2$** in CH_3CN/[^{n}Bu$_4$N][PF$_6$] and d) oxidative spectroelectrochemistry (red → blue; λ_{max} = 320 nm, λ_{isosb} = 355 nm) and e) reductive spectroelectrochemistry (red → blue; λ_{max} = 430, 750, 860 nm, λ_{isosb} = 401, 590 nm) of **[V(tpe)$_2$]Cl$_2$** in CH_3CN/[^{n}Bu$_4$N][PF$_6$].

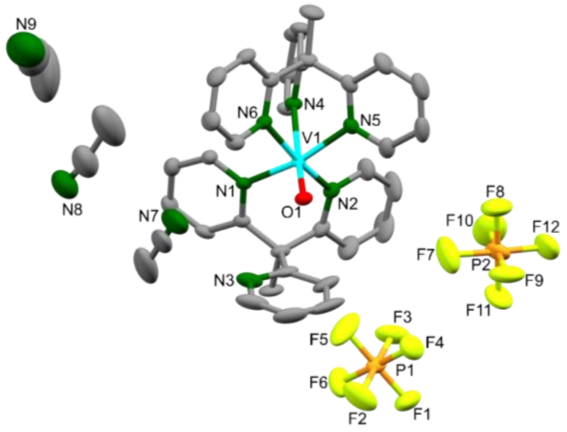

Figure S8. Structure of **[VO(κ^2-tpe)(tpe)][PF$_6$]$_2$×3CH$_3$CN** with thermal ellipsoids set to 50 % probability. CH hydrogen atoms omitted.

Table S4. Bond lengths [Å] and angles [deg] of **[VO(κ^2-tpe)(tpe)][PF$_6$]$_2$×3CH$_3$CN** from single crystal XRD measurement.

bond lengths		bond angles	
V1–N1	2.1153(39)	N1–V1–O1	99.98(16)
V1–N2	2.1215(39)	N1–V1–N2	83.48(16)
V1···N3	4.04	N1–V1–N4	85.41(14)
V1–N4	2.2431(35)	N1–V1–N5	166.03(15)
V1–N5	2.1204(40)	N1–V1–N6	94.52(16)
V1–N6	2.0996(39)	N2–V1–O1	99.02(16)
V1–O1	1.5927(32)	N2–V1–N4	86.03(15)
		N2–V1–N5	96.39(17)
		N2–V1–N6	168.06(15)
		N4–V1–O1	172.96(15)
		N4–V1–N5	80.65(15)
		N4–V1–N6	82.08(15)
		N5–V1–N6	82.72(16)
		N5–V1–O1	93.84(16)

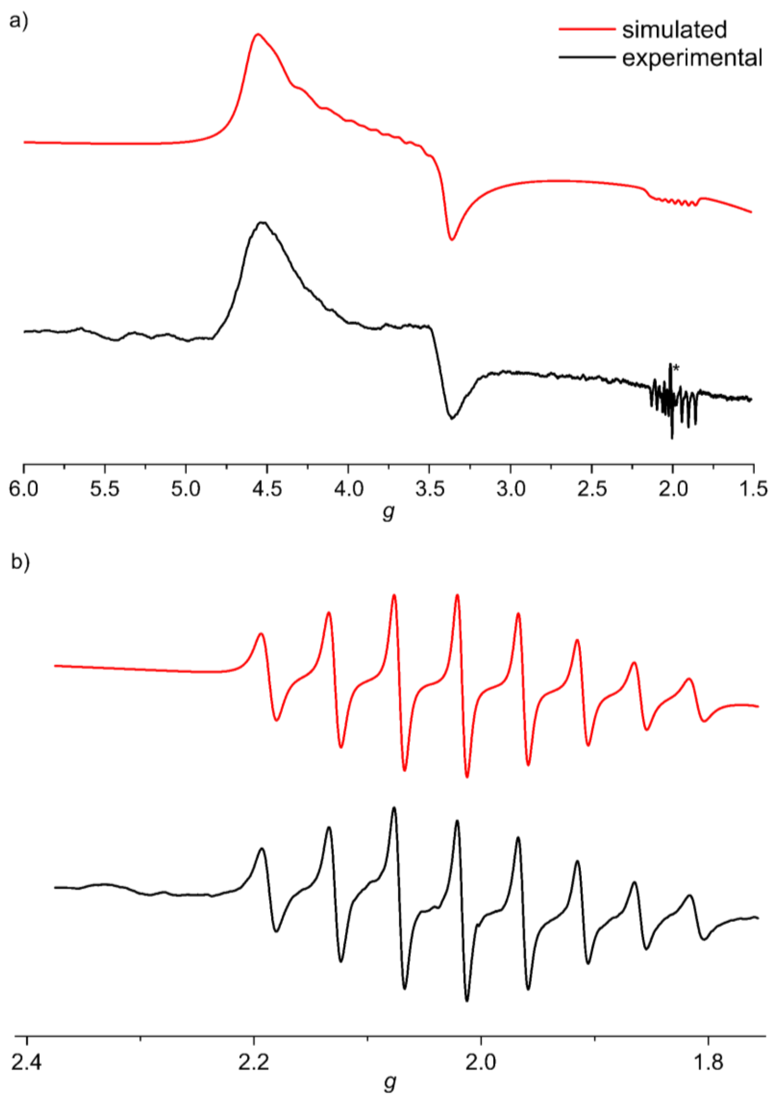

Figure S9. a) X-band cw-EPR spectrum of **[V(tpe)$_2$]Cl$_2$** in ethanol/methanol (*v*:*v*, 3:2) at 77 K (black line) (the asterisk * denotes a trace impurity) and simulated spectrum (red) using $g_{1,2,3}$ = 3.957, 3.841, 1.983; $A_{1,2,3}$(^{51}V)= 361.7, 354.2, 186.8 MHz; peak-to-peak Gaussian/Lorentzian line widths = 1.731/2.720 mT. These data are comparable to literature data of vanadium(II) complexes.[30] b) X-band cw-EPR spectrum of **[VO(κ^2-tpe)(tpe)][PF$_6$]$_2$×3CH$_3$CN** in CH$_3$CN at 293 K (black) and simulated spectrum (red) with tumbling spin systems in the slow-motional regime using $g_{1,2,3}$ = 1.9736, 1.9760, 1.9840; $A_{1,2,3}$(^{51}V) = 287.7, 244.8, 222.6 MHz; peak-to-peak Gaussian/Lorentzian line widths = 0.933/0.408 mT and rotational correlation time τ_{corr} = 10^{-9} s; g_{av} = 1.9778; A_{av}(^{51}V) = 255 MHz. These data are comparable to literature data of vanadyl(VI) complexes.[31]

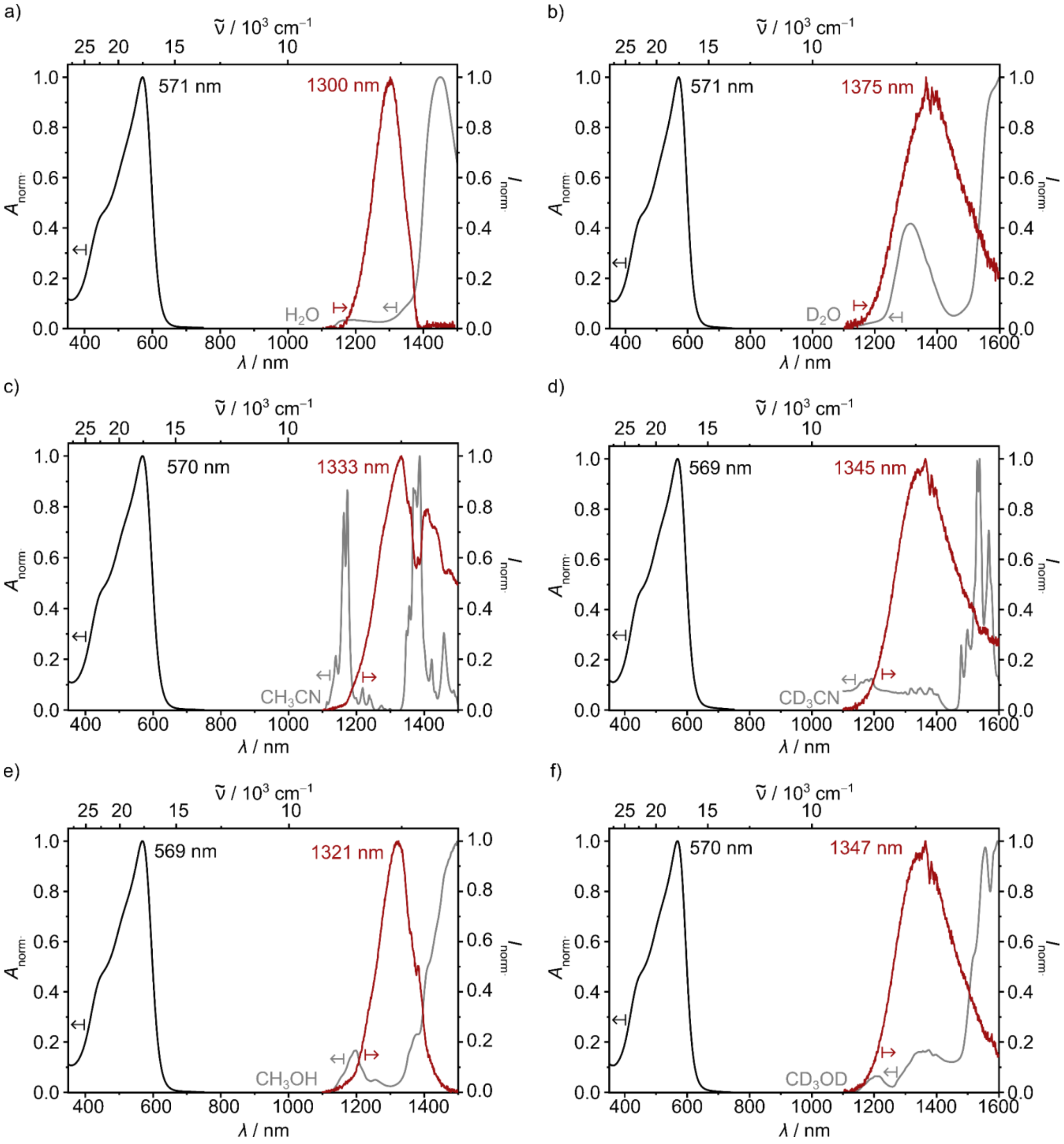

Figure S10. Absorption (black) and emission spectra (red) of **[V(tpe)$_2$]Cl$_2$** with λ_{exc} = 450 nm in a) H_2O, b) D_2O, c) CH_3CN, d) CD_3CN, e) CH_3OH and f) CD_3OD. The normalized NIR absorption spectra of the respective solvents are displayed in gray. Apparent emission maxima are labeled in red.

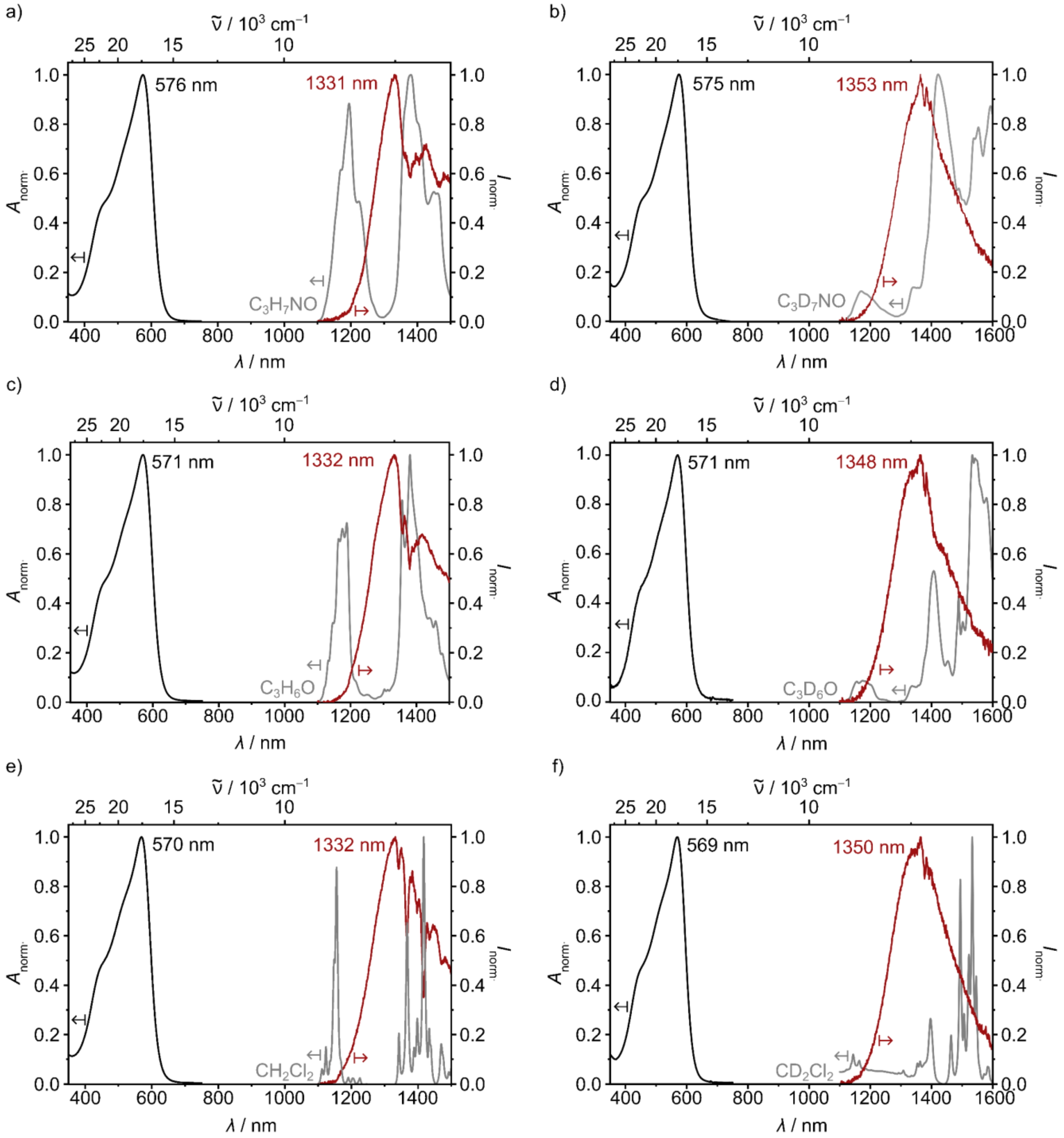

Figure S11. Absorption (black) and emission spectra (red) of **[V(tpe)$_2$]Cl$_2$** with λ_{exc} = 450 nm in a) dimethylformamide (C_3H_7NO), b) deuterated dimethylformamide (C_3D_7NO), c) acetone (C_3H_6O), d) deuterated acetone (C_3D_6O), e) CH_2Cl_2 and f) CD_2Cl_2. The normalized NIR absorption spectra of the respective solvents are displayed in gray. Apparent emission maxima are labeled in red.

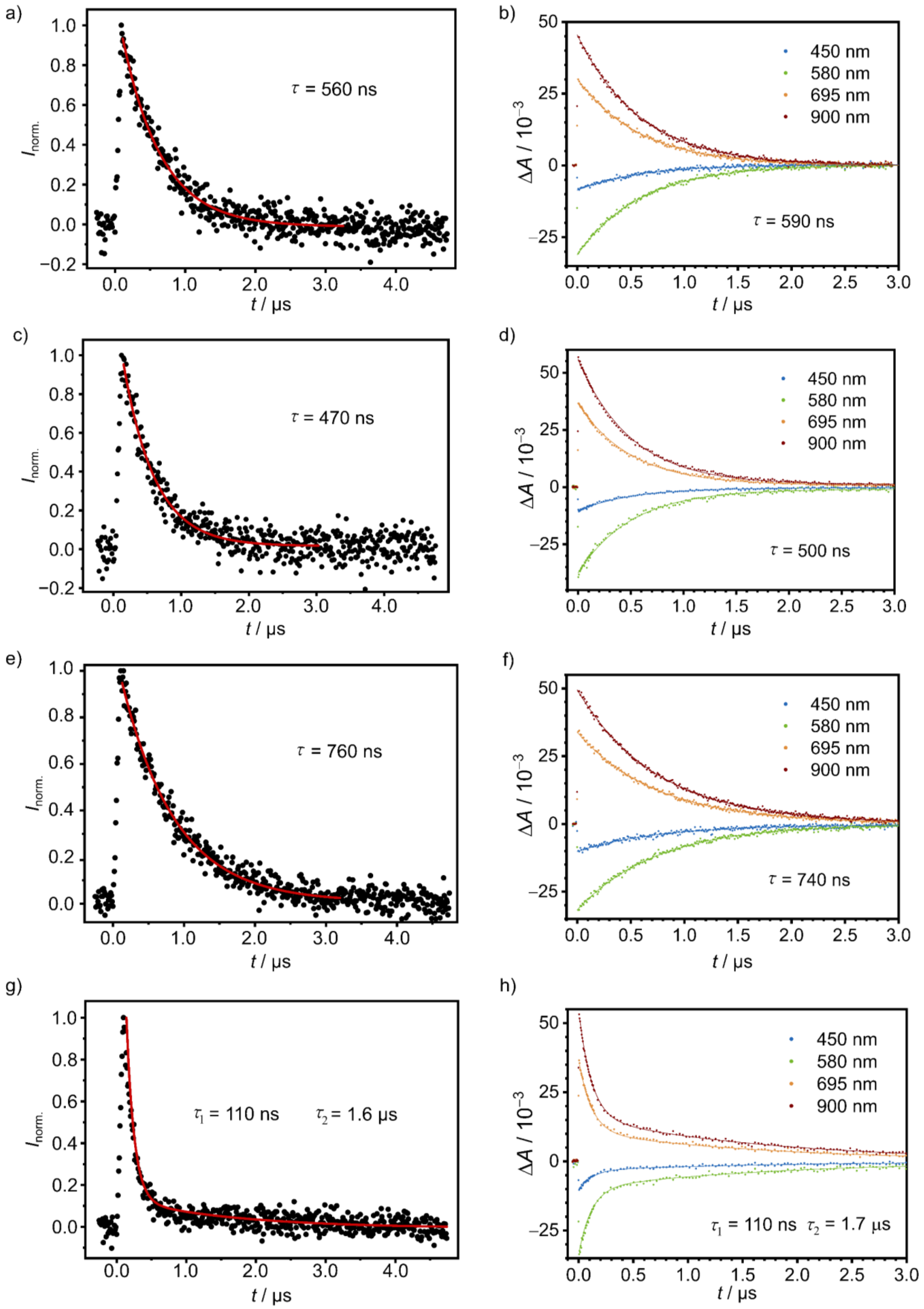

Figure S12. Luminescence decay traces of **[V(tpe)$_2$]Cl$_2$** in a) D_2O/Ar, c) D_2O/air, e) CD_3CN/Ar and g) CD_3CN/air (left panels) with λ_{exc} = 450 nm (cw laser) and decay traces from ns-transient absorption spectroscopy at selected wavelengths b) D_2O/Ar, d) D_2O/air, f) CD_3CN/Ar and h) CD_3CN/air (right panels) with λ_{exc} = 570 nm at 293 K. All fits to the data were monoexponential, except in CD_3CN/air (panels g and h), where an excited state equilibrium with $^1O_2$ is established and a biexponential decay results (see below).

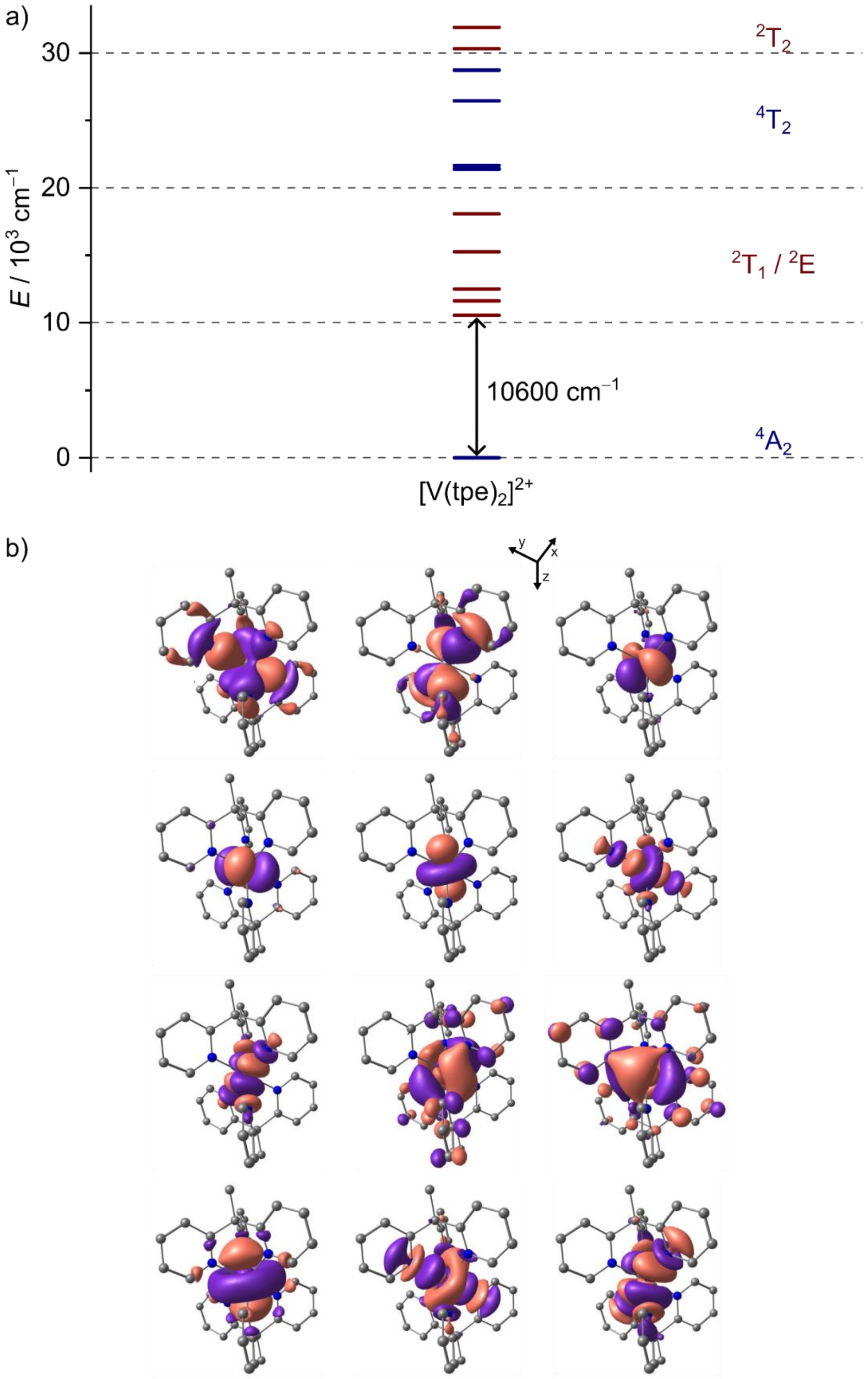

Figure S13. a) Energy levels of quartet (blue) and doublet (red) states from CASSCF(7,12)-SC-NEVPT2 calculations of DFT-optimized **$[V(tpe)_2]^{2+}$** and b) orbitals used in the CASSCF(7,12)-SC-NEVPT2 calculation of **$[V(tpe)_2]^{2+}$**, depicted at a contour value of 0.03 a.u. (hydrogen atoms omitted for clarity).

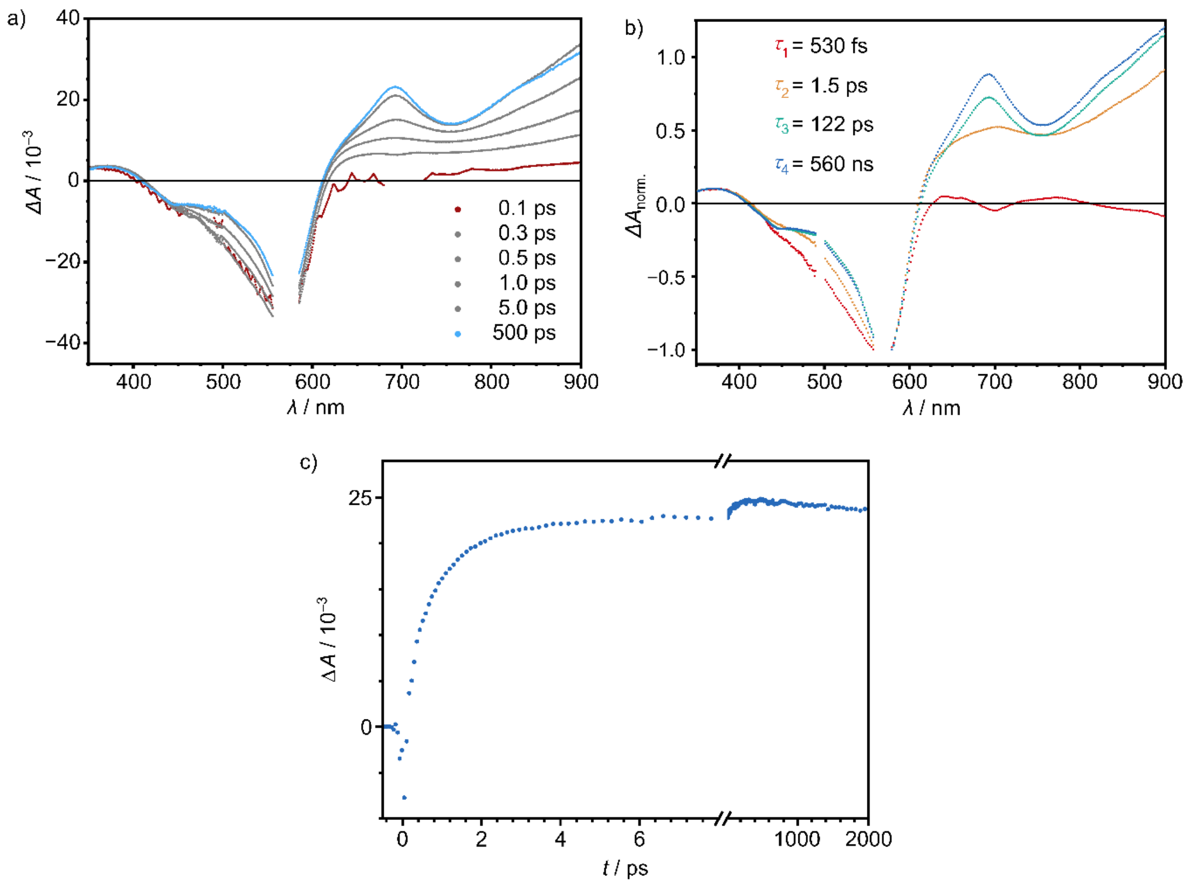

Figure S14. a) fs-Transient absorption spectra and b) evolution-associated difference spectra (EADS) of **[V(tpe)$_2$]Cl$_2$** in D_2O after excitation with 570 nm pulses at 293 K. c) Kinetic trace at the band maximum of the fs-transient absorption spectra at 690 nm in H_2O/Ar showing a fast increase followed by a slower decrease of the band.

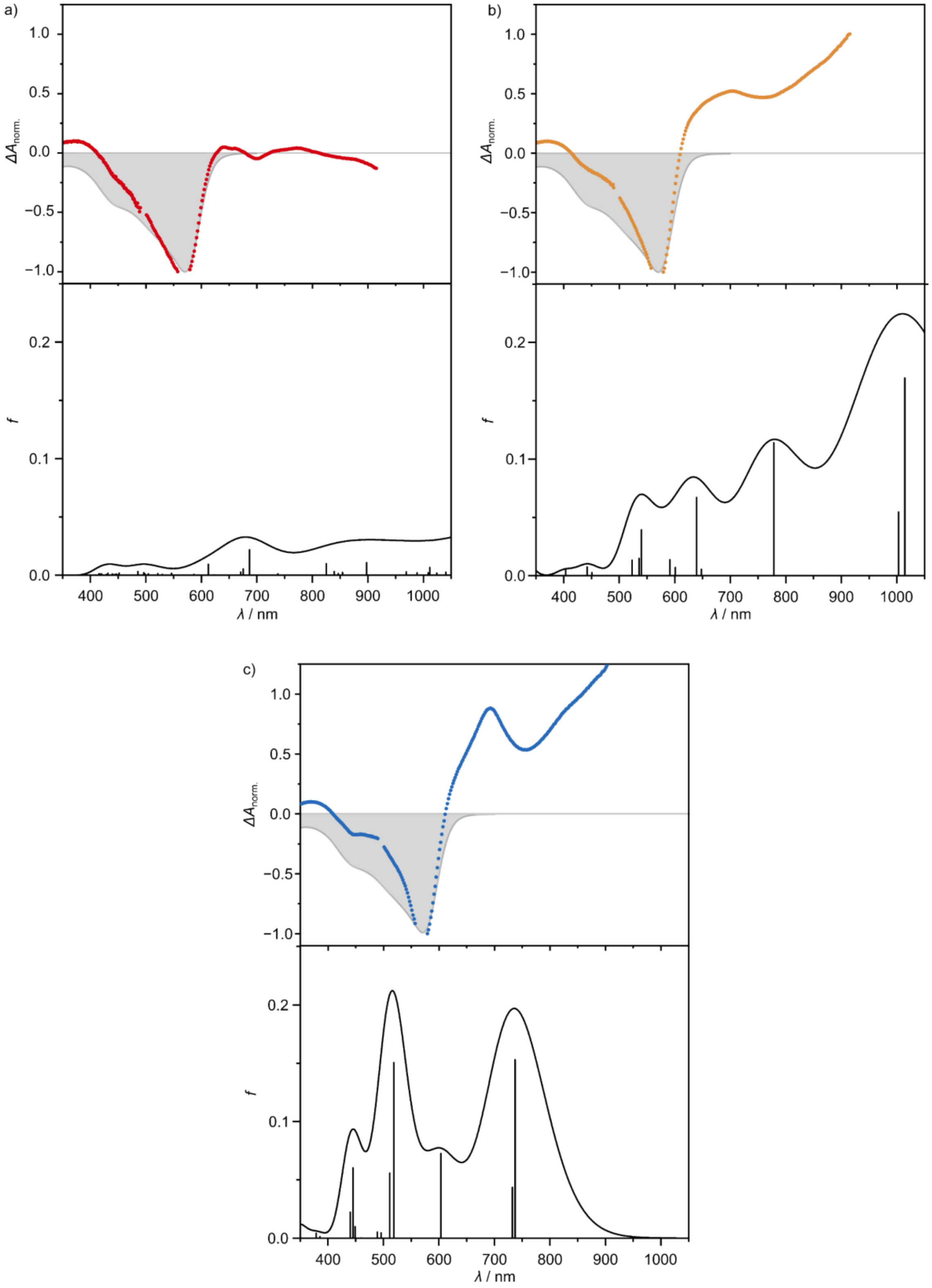

Figure S15. Top, EADS of the first (a, red), second (b, orange) and fourth (c, blue) component of the global fit of the fs-transient absorption spectra shown in Fig. S14 superimposed with the ground state bleach (gray area). Bottom, the DFT simulated transient absorption spectra (oscillator strength *f*) of the excited quartet state (root 1) (a), excited doublet state (root 1) (b) and the TD-DFT calculated transitions of the lowest energy doublet state (c) as well as the envelope band shape composed of Gaussians with FWHM of 2250 cm^{-1} (black line).

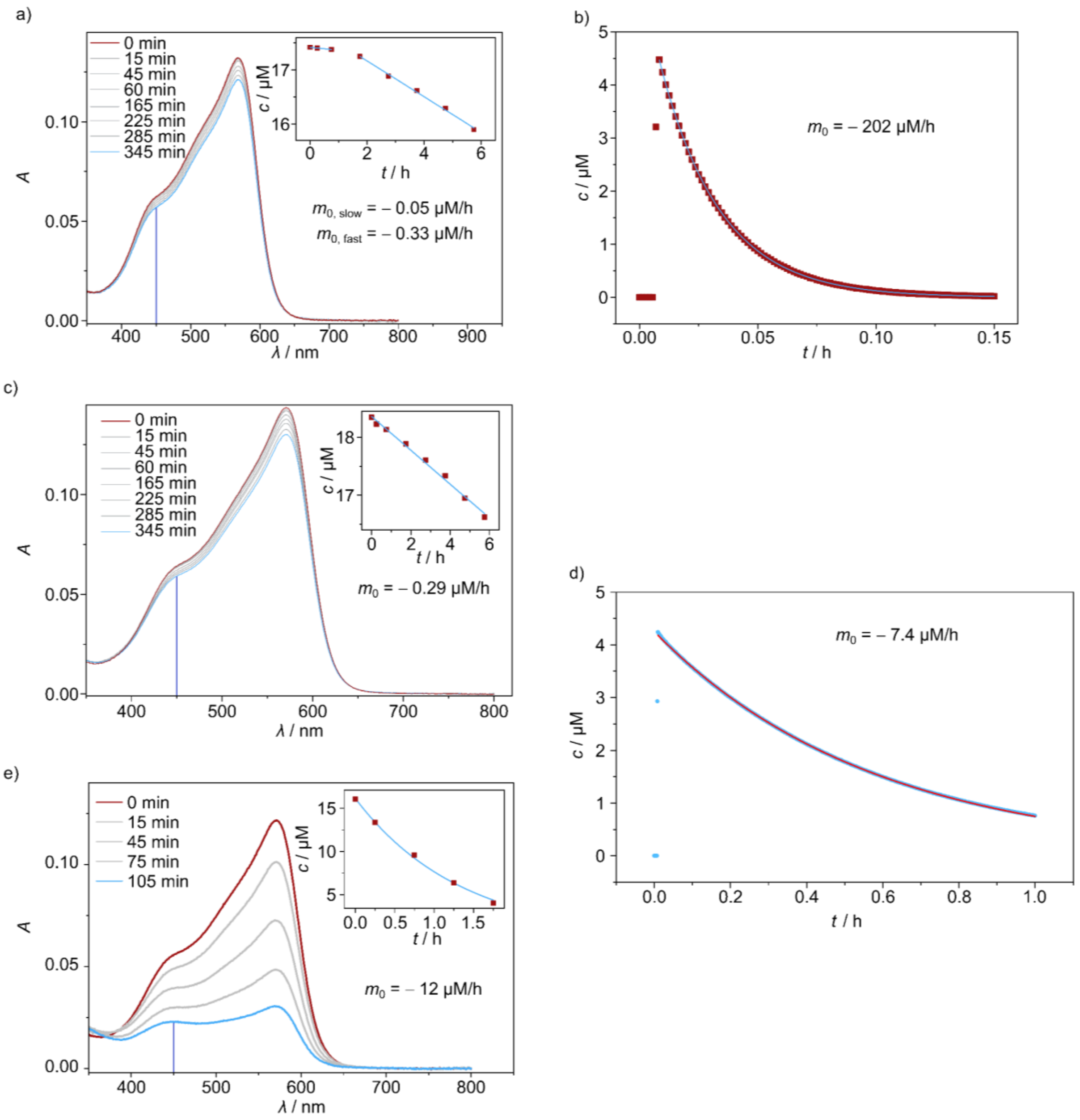

Figure S16. Photostability experiments of **[V(tpe)$_2$]Cl$_2$** (left) and [Ru(bpy)$_3$]Cl$_2$ (right) in a) deaearated acetonitrile, c) in deaerated water and e) in aerated water with an excitation of 450 nm. Main plot of a) and c) show absorption spectra after irradiating the **[V(tpe)$_2$]Cl$_2$** solution for 0 min (red), 15 min, 45 min, 60 min, 165 min, 225 min, 285 min and 345 min (light blue) (dark blue vertical line indicates excitation wavelength). Main plot of e) shows absorption spectra after irradiating the **[V(tpe)$_2$]Cl$_2$** solution for 0 min (red), 15 min, 45 min, 75 min and 105 min (light blue) (dark blue vertical line indicates excitation wavelength). Insets of a), c) and e) and main plots of b) and d) show concentration vs. time plots (red). The concentration vs. time plots are superimposed with linear fits for a) and c) (blue, $c = m_{0,V}\ t + c_0$) and with monoexponential fits for b), d) and e) (blue, $c = c_0\ e^{-k_{deg}t}$). The initial degradation rate $m_{0,Ru}$ is given by the product of the initial concentration c_0 and the degradation rate constant k_{deg}.

Table S5. Relative photodegradation quantum yields $\phi_{rel} = \frac{\phi_{deg,V}}{\phi_{deg,Ru}}$ and stability factors $f_{stab} = \frac{\phi_{deg,Ru}}{\phi_{deg,V}}$ of $[Ru(bpy)_3]Cl_2$ and **$[V(tpe)_2]Cl_2$** in deaerated acetonitrile, deaerated water and in aerated water.

	acetonitrile / Ar		water / Ar		water / air
	**$[Ru(bpy)_3]Cl_2$**	**$[V(tpe)_2]Cl_2$** [a]	**$[Ru(bpy)_3]Cl_2$**	**$[V(tpe)_2]Cl_2$**	**$[V(tpe)_2]Cl_2$**
$\dot{N}_{deg}$/ s^{-1}	$1.0 \cdot 10^{14}$	$1.7 \cdot 10^{11}$	$3.7 \cdot 10^{12}$	$1.5 \cdot 10^{11}$	$6.0 \cdot 10^{12}$
$\dot{N}_{abs}$ / s^{-1}	$3.0 \cdot 10^{17}$	$2.9 \cdot 10^{17}$	$2.8 \cdot 10^{17}$	$2.9 \cdot 10^{17}$	$2.6 \cdot 10^{17}$
$\phi_{deg,x}$ / %	$3.4 \cdot 10^{-2}$	$5.8 \cdot 10^{-5}$	$1.3 \cdot 10^{-3}$	$5.0 \cdot 10^{-5}$	$2.3 \cdot 10^{-3}$
ϕ_{rel}	1	$1.7 \cdot 10^{-3}$	1	$3.8 \cdot 10^{-2}$	1.8
f_{stab}	1	590	1	26	0.6

[a] The slope after the induction period was used for the calculations (starting at 1.75 h, see Fig. S16a).

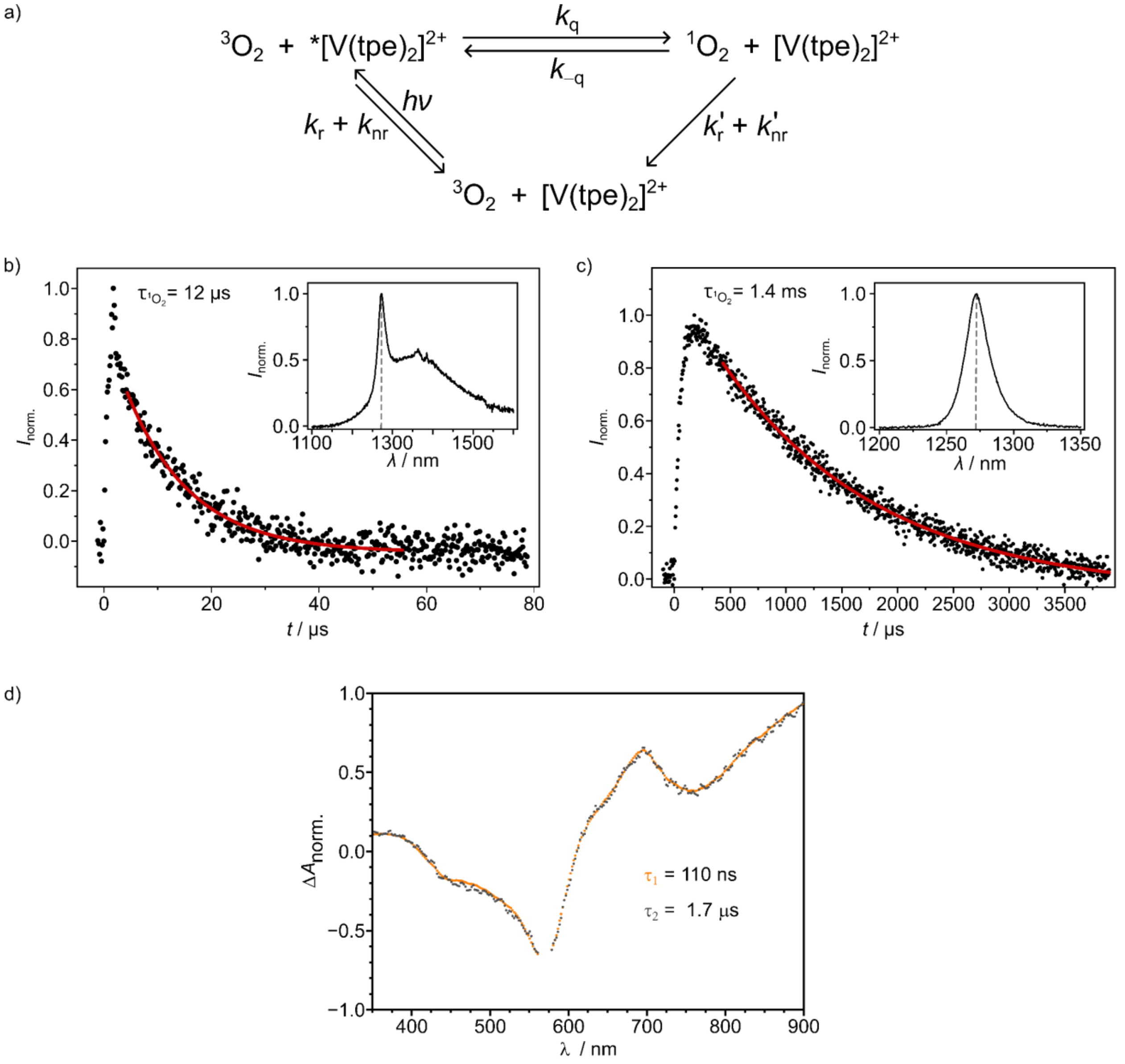

Figure S17. a) Kinetic scheme of the excited state energy transfer between **[V(tpe)$_2$]$^{2+}$** and O_2 in CD_3CN. Emission spectra of $^1O_2$ obtained by excitation of b) **[V(tpe)$_2$]Cl$_2$** in air-saturated CD_3CN with 450 nm (cw laser) and c) [Cr(ddpd)$_2$][PF$_6$]$_3$ [34] as sensitizer in air-saturated CD_3CN with a xenon lamp at 430 nm. Main plots show decay traces of the emission at 1270 nm (gray data points) with tail fit (red) overlaid on the data. The insets show the corresponding emission spectra. In panel b) emission bands of $^1O_2$ (sharp) and **[V(tpe)$_2$]$^{2+}$** (broad) are observed simultaneously. d) EADS of ***[V(tpe)$_2$]$^{2+}$** before and after establishing the excited state equilibrium in air-saturated CD_3CN. The EADS are virtually identical.

Kinetic modeling of the excited state equilibrium of $^1O_2$ and $^*[V(tpe)_2]^{2+}$

In air-saturated acetonitrile, a biexponential decay of the long-lived excited state $^*[V(tpe)_2]^{2+}$ is observed upon laser excitation (Fig. S12g and S12h). This biexponential decay kinetics can be rationalized with an excited state equilibrium between $^*[V(tpe)_2]^{2+}$ and $^1O_2$, which is illustrated in Fig. S17a. The establishment of this equilibrium is much faster than the photophysical deactivation of both $^*[V(tpe)_2]^{2+}$ and $^1O_2$. Hence, the time derivative of the $^*[V(tpe)_2]^{2+}$ concentration can be expressed with a simplified differential rate law (eq. S4).

$$\frac{d[\,^*[\mathrm{V(tpe)_2}]^{2+}]}{dt} = -\,k_q\,[\,^3O_2][\,^*[\mathrm{V(tpe)_2}]^{2+}] + k_{-q}[\,^1O_2][[\mathrm{V(tpe)_2}]^{2+}] \qquad \text{(S4)}$$

For such a system of two equilibrating states, the time needed to establish the equilibrium τ_{eq} is given by:

$$\tau_{\mathrm{eq}} = \left(k_{\mathrm{q}}[\,^3O_2] + k_{-q}[[\mathrm{V(tpe)_2}]^{2+}]\right)^{-1} \qquad \text{(S5)}$$

After reaching equilibrium, $^*[V(tpe)_2]^{2+}$ and $^1O_2$ have a fixed ratio, which is governed by the ratio of the forward and backward energy transfer rates k_q and k_{-q}.

$$\frac{[\,^*[\mathrm{V(tpe)_2}]^{2+}]}{[\,^1O_2]} = const. = \frac{k_{-q}[[\mathrm{V(tpe)_2}]^{2+}]}{k_{\mathrm{q}}[\,^3O_2]} \qquad \text{(S6)}$$

The total concentration of both equilibrated excited states $[\,^*E]$ equals:

$$[\,^*E] = [\,^*[\mathrm{V(tpe)_2}]^{2+}] + [\,^1O_2] \qquad \text{(S7)}$$

With the decay rates $k_d = k_r + k_{nr}$ and $k'_d = k'_r + k'_{nr}$ (Fig. S17a), the decay of the equilibrated states can be expressed with eq. S8.

$$\frac{d[\,^*E]}{dt} = -k_d[\,^*[\mathrm{V(tpe)_2}]^{2+}] - k'_d[\,^1O_2] \qquad \text{(S8)}$$

Combining eq. S6 and eq. S7, the concentration of $^*[V(tpe)_2]^{2+}$ can be written as eq. S9:

$$[\,^*[\mathrm{V(tpe)_2}]^{2+}] = [\,^*E]\frac{k_{-q}[[\mathrm{V(tpe)_2}]^{2+}]}{k_{\mathrm{q}}[\,^3O_2] + k_{-q}[[\mathrm{V(tpe)_2}]^{2+}]} \qquad \text{(S9)}$$

With the assumption that $k'_d[\,^1O_2] \approx 0$ ($\tau_{^1O_2}$= 1.4 ms, Fig. S17c), a first order differential rate law for the decay of $[\,^*E]$ is obtained (eq. S10).

$$\frac{d[\,^*E]}{dt} = -\frac{k_d \cdot k_{-q}[[\mathrm{V(tpe)_2}]^{2+}]}{k_{\mathrm{q}}[\,^3O_2] + k_{-q}[[\mathrm{V(tpe)_2}]^{2+}]}[\,^*E] \qquad \text{(S10)}$$

Integration of eq. S10 yields eq. S11, which describes the decay of both equilibrated excited states with the corresponding lifetime τ_E.

$$[\,^*E] = [\,^*E]_0 e^{-\frac{k_d \cdot k_{-q}[[\mathrm{V(tpe)_2}]^{2+}]}{k_{\mathrm{q}}[\,^3O_2]+k_{-q}[[\mathrm{V(tpe)_2}]^{2+}]}t} \qquad \text{(S11)}$$

$$\tau_E = \frac{k_{\mathrm{q}}[\,^3O_2] + k_{-q}[[\mathrm{V(tpe)_2}]^{2+}]}{k_d \cdot k_{-q}[[\mathrm{V(tpe)_2}]^{2+}]} \qquad \text{(S12)}$$

With $[^3O_2]$ = 1.9 mM in acetonitrile,[35] $[[V(tpe)_2]^{2+}]$ = 0.82 mM and k_d = 1/750 ns, eq. S12 yields the ratio between k_q and k_{-q}, which amounts to 0.55. This ratio indicates that the quenching of $^*[V(tpe)_2]^{2+}$ by $^3O_2$ is slightly uphill and confirms the energetical proximity of $^*[V(tpe)_2]^{2+}$ and $^1O_2$ excited states. Insertion of k_q/k_{-q} and τ_{eq}= 110 ns (Fig. S12g and S12h) in eq. S5 yields the absolute values of the rate constants of the forward and backward energy transfer as $k_q = 2.7\times10^9$ M^{-1} s^{-1} and $k_{-q} = 4.9\times10^9$ M^{-1} s^{-1}.

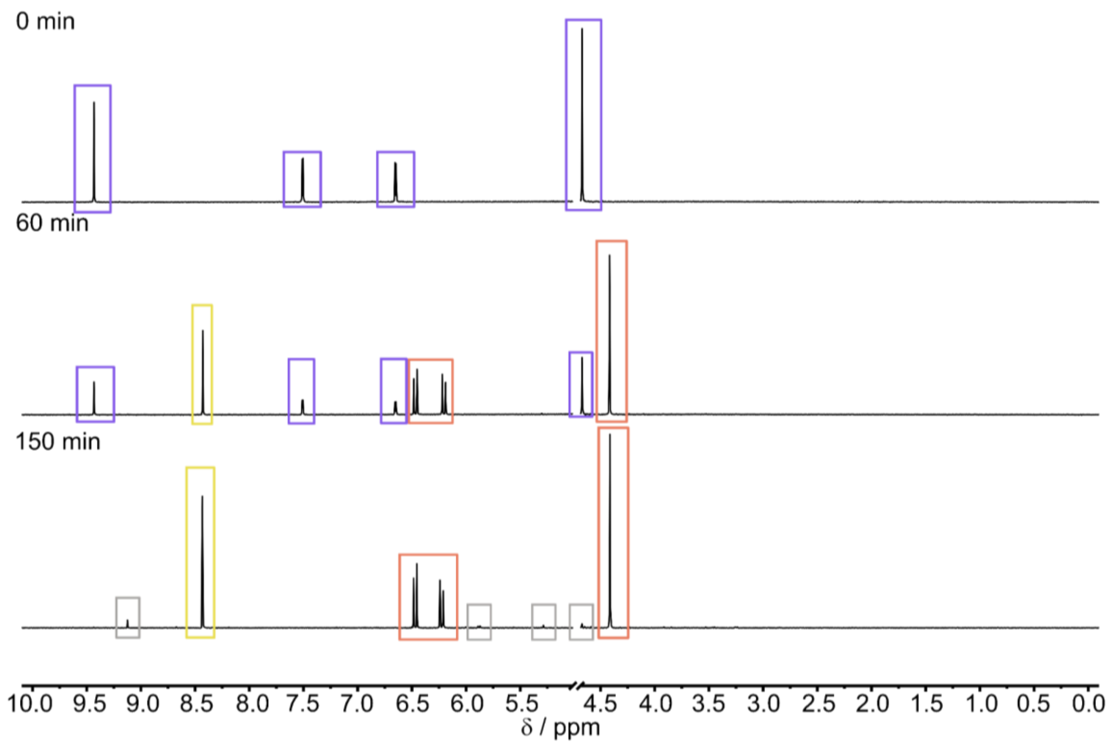

Figure S18. ^{1}H NMR spectra during the photocatalytic conversion of 5-HMF (25 mM) in D_2O/phosphate buffer (Na_2HPO_4/NaH_2PO_4 100 mM/10 mM) at 278 K using a 560 nm UHP LED (set to 50 % intensity) and **[V(tpe)$_2$]Cl$_2$** (0.1 mM) as photosensitizer. Resonances of 5-HMF (purple), (*Z*)-5-hydroxy-4-keto-2-pentenoate (orange) and formiate (yellow) are indicated by the respective colors. A side product (gray) forms from the product in addition in small amounts (< 8% at 150 min).

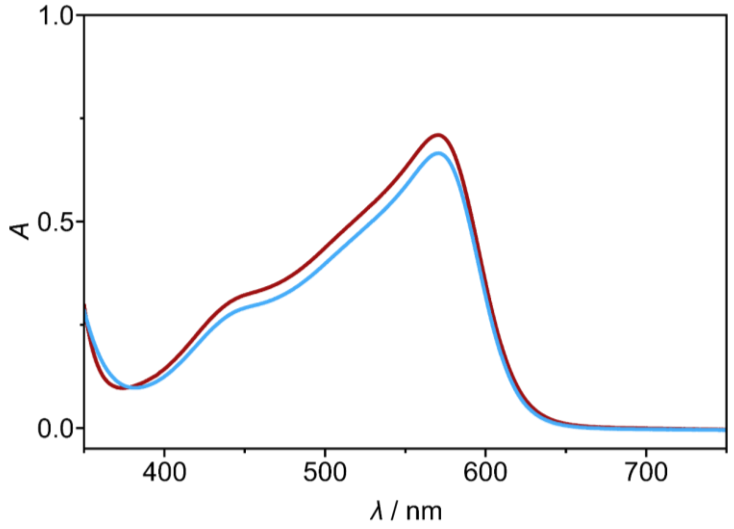

Figure S19. UV/vis absorption spectra of **[V(tpe)$_2$]Cl$_2$** in D_2O/phosphate buffer before (red) and after (blue) the photooxidation of 5-HMF (Fig. S18).

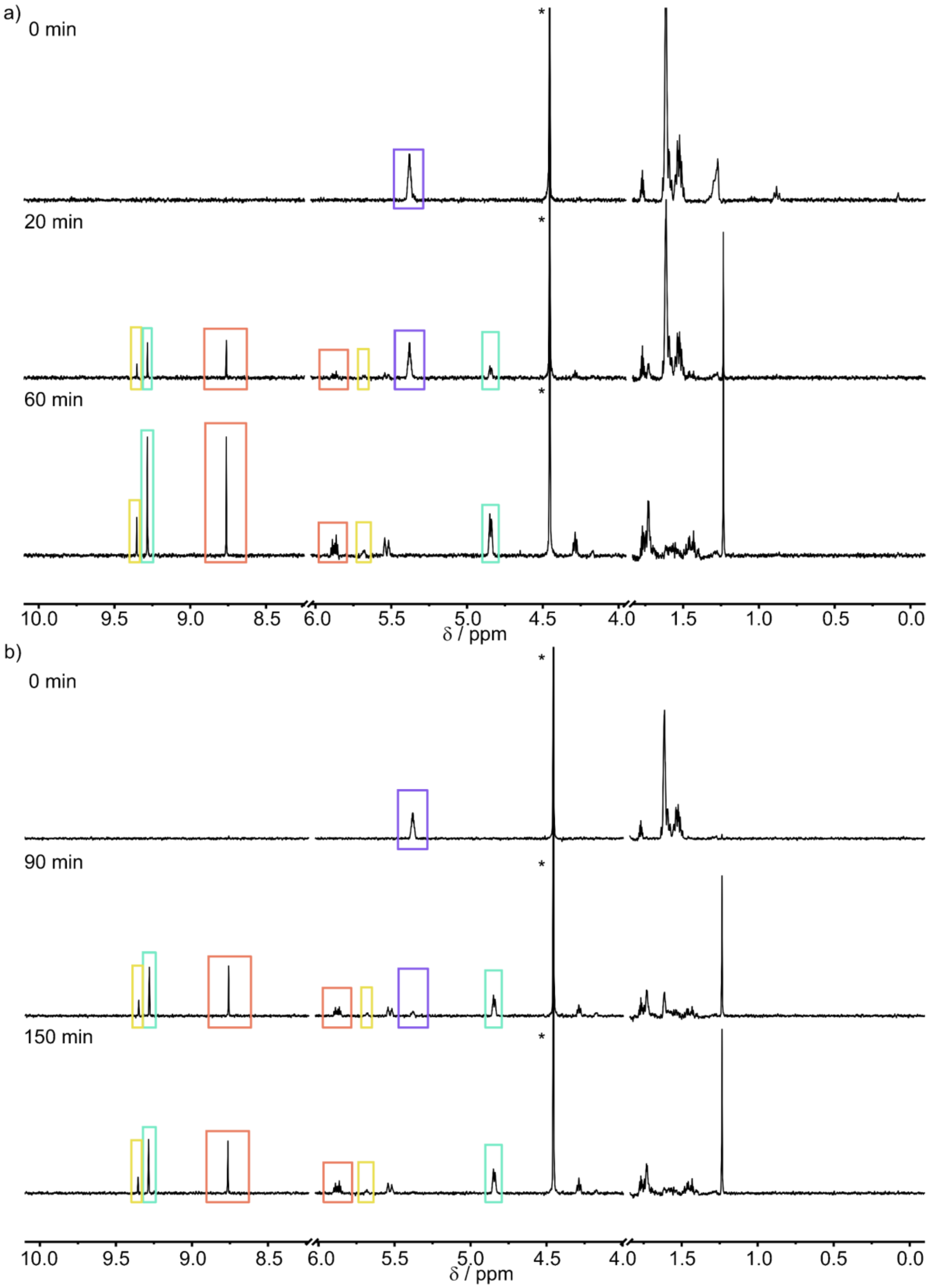

Figure S20. Partial ^{1}H NMR spectra during the photocatalytic conversion of 1-MCH (25 mM) in CD_3CN at the indicated times using a) a 560 nm UHP LED (set to 50 % intensity) and **[V(tpe)$_2$]Cl$_2$** (0.1 mM) as photosensitizer and b) a 625 nm UHP LED (set to 50 % intensity) and **[V(tpe)$_2$]Cl$_2$** (0.25 mM) as photosensitizer. Resonances of 1-MCH (purple) and the products 3-hydroperoxy-3-methylcyclohex-1-ene (orange), 2-methyl-cyclohex-2-enyl hydroperoxide (yellow) and 1-hydroperoxy-2-methylenecyclohexane (green) are indicated by the respective colors. Ethylene carbonate was used as internal standard and is indicated by an asterisk (*).

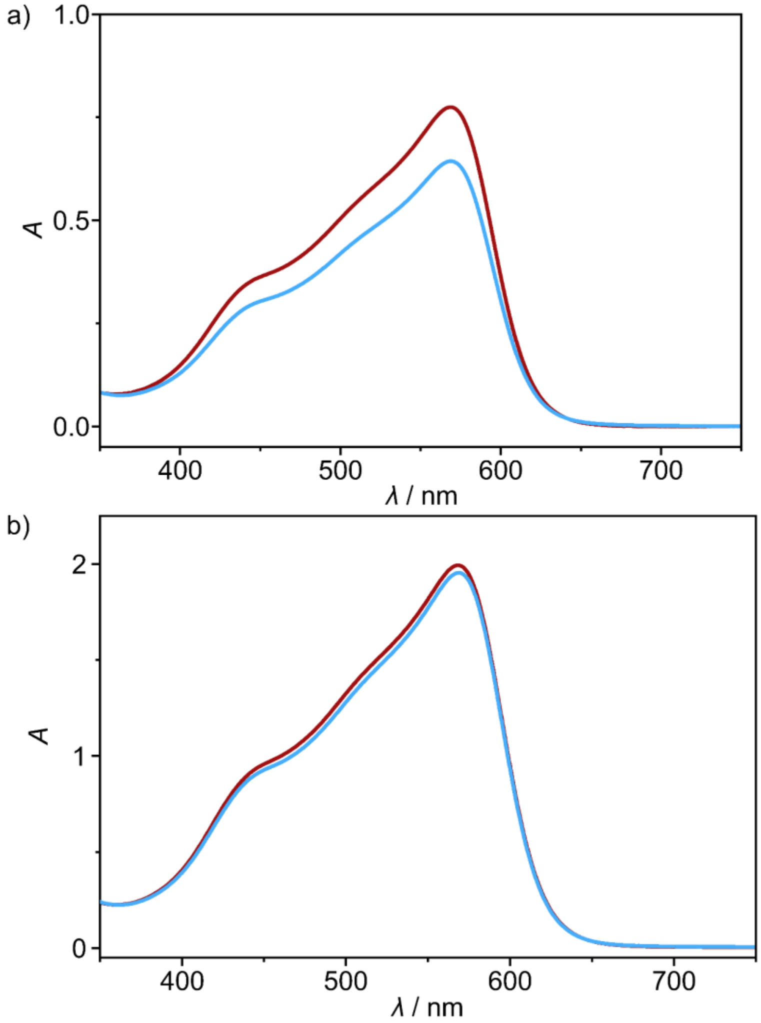

Figure S21. UV/vis absorption spectra of **[V(tpe)$_2$]Cl$_2$** before (red) and after (after) the photooxidation of 1-MCH with a a) 560 nm UHP-LED lamp (set to 50 % intensity) and b) 625 nm UHP-LED (set to 50 % intensity) (Fig. S20).

References

(1) Santoro, A.; Sambiagio, C.; McGowan, P. C.; Halcrow, M. A. Synthesis and coordination chemistry of 1,1,1-tris-(pyrid-2-yl)ethane. *Dalton Trans.* **2015**, *44*, 1060–1069.

(2) Anderson, S. J.; Wells, F. J.; Wilkinson, G.; Hussain, B.; Hursthouse, M. B. 1,2-Bis(dimethyl)phosphinoethane complexes of molybdenum and vanadium. X-ray crystal structure of trans-[MoCl(η^2-NCMe)(dmpe)$_2$]BPh$_4$, trans-[(SPh)$_2$(dmpe)$_2$], trans-[V(NCMe)$_2$(dmpe)$_2$]BPh$_4$, trans-[V(CNBut)$_2$(dmpe)$_2$](PF$_6$)$_2$. *Polyhedron* **1988**, *7*, 2615–2626.

(3) Stoll, S.; Schweiger, A. EasySpin, a comprehensive software package for spectral simulation and analysis in EPR. *J. Magn. Reson.* **2006**, *178*, 42–55.

(4) Fulmer, G. R.; Miller, A. J. M.; Sherden, N. H.; Gottlieb, H. E.; Nudelman, A.; Stoltz, B. M.; Bercaw, J. E.; Goldberg, K. I. NMR Chemical Shifts of Trace Impurities: Common Laboratory Solvents, Organics, and Gases in Deuterated Solvents Relevant to the Organometallic Chemist. *Organometallics* **2010**, *29*, 2176−2179.

(5) Müller, C.; Pascher, T.; Eriksson, A.; Chabera, P.; Uhlig, J. KiMoPack: A python Package for Kinetic Modeling of the Chemical Mechanism. *J. Phys. Chem. A* **2022**, *126*, 4087–4099.

(6) STOE & Cie, X-Area; STOE & Cie GmbH: Darmstadt, Germany.

(7) Blessing, R. H. An empirical correction for absorption anisotropy. *Acta Crystallogr. A* **1995**, *51*, 33–38.

(8) Spek, A. L. Structure validation in chemical crystallography. *Acta Crystallogr. D* **2009**, *65*, 148–155.

(9) Sheldrick, G. M. SHELXT - integrated space-group and crystal-structure determination. *Acta Crystallogr., Sect. A* **2015**, *71*, 3–8.

(10) Sheldrick, G. M. Crystal structure refinement with SHELXL. *Acta Crystallogr., Sect. C* **2015**, *71*, 3–8.

(11) Hübschle, C. B.; Sheldrick, G. M.; Dittrich, B. ShelXle: a Qt graphical user interface for SHELXL. *J. Appl. Crystallogr.* **2011**, *44*, 1281−1284.

(12) Neese, F. Software update: The ORCA program system – Version 5.0. *WIREs Comput. Mol. Sci.* **2022**, *12*, No. e1606.

(13) Neese, F.; Wennmohs, F.; Becker, U.; Riplinger, C. The ORCA quantum chemistry program package. *J. Chem. Phys.* **2020**, *152*, 224108.

(14) Becke, A. D. Density−functional thermochemistry. III. The role of exact exchange. *Chem. Phys.* **1993**, *98*, 5648−5652.

(15) Miehlich, B.; Savin, A.; Stoll, H.; Preuss, H. Results obtained with the correlation energy density functionals of Becke and Lee. Yang and Parr. *Chem. Phys. Lett.* **1989**, *157*, 200−206.

(16) Weigend, F.; Ahlrichs, R. Balanced basis sets of split valence, triple zeta valence and quadruple zeta valence quality for H to Rn: Design and assessment of accuracy. *Phys. Chem. Chem. Phys.* **2005**, *7*, 3297−3305.

(17) Weigend, F. Accurate Coulomb-fitting basis sets for H to Rn. *Phys. Chem. Chem. Phys.* **2006**, *8*, 1057−1065.

(18) Pantazis, D. A.; Chen, X.-Y.; Landis, C. R.; Neese, F. All-Electron Scalar Relativistic Basis Sets for Third-Row Transition Metal Atoms. *J. Chem. Theory Comput.* **2008**, *4*, 908−919.

(19) Neese, F.; Wennmohs, F.; Hansen, A.; Becker, U. Efficient, approximate and parallel Hartree−Fock and hybrid DFT calculations. A 'chain-of-spheres' algorithm for the Hartree−Fock exchange. *Chem. Phys.* **2009**, *356*, 98−109.

(20) Izsák, R.; Neese, F. An overlap fitted chain of spheres exchange method. *J. Chem. Phys.* **2011**, *135*, 144105.

(21) Barone, V.; Cossi, M. Quantum Calculation of Molecular Energies and Energy Gradients in Solution by a Conductor Solvent Model. *J. Phys. Chem. A* **1998**, *102*, 1995−2001.

(22) Miertuš, S.; Scrocco, E.; Tomasi, J. Electrostatic interaction of a solute with a continuum. A direct utilizaion of ab initio molecular potentials for the prevision of solvent effects. *Chem. Phys.* **1981**, *55*, 117−129.

(23) Grimme, S.; Antony, J.; Ehrlich, S.; Krieg, H. A consistent and accurate ab initio parametrization of density functional dispersion correction (DFT-D) for the 94 elements H-Pu. *J. Chem. Phys.* **2010**, *132*, 154104.

(24) Grimme, S.; Ehrlich, S.; Goerigk, L. Effect of the damping function in dispersion corrected density functional theory. *J. Comput. Chem.* **2011**, *32*, 1456−1465.

(25) Plasser, F. TheoDORE: A toolbox for a detailed and automated analysis of electronic excited state computations. *J. Chem. Phys.* **2020**, *152*, No. 084108.

(26) Neese, F. Prediction of molecular properties and molecular spectroscopy with density functional theory: From fundamental theory to exchange-coupling. *Coord. Chem. Rev.* **2009**, *253*, 526–563.

(27) Atanasov, M.; Ganyushin, D.; Sivalingam, K.; Neese, F. A Modern First-Principles View on Ligand Field Theory Through the Eyes of Correlated Multireference Wavefunctions. *Struct. Bond.* **2012**, *143*, 149–220.

(28) Förster, C.; Heinze, K. The Photophysics and Applications of Molecular Rubies. *Adv. Inorg. Chem.* **2024**, *83*, 111–159.

(29) Reichenauer, F.; Wang, C.; Förster, C.; Boden, P.; Ugur, N.; Báez-Cruz, R.; Kalmbach, J.; Carrella, L. M.; Rentschler, E.; Ramanan, C.; Niedner-Schatteburg, G.; Gerhards, M.; Seitz, M.; Resch-Genger, U.; Heinze, K. Strongly Red-Emissive Molecular Ruby $[Cr(bpmp)_2]^{3+}$ surpasses $[Ru(bpy)_3]^{2+}$. *J. Am. Chem. Soc.* **2021**, *143*, 11843–11855.

(30) Scott, M. J.; Wilisch, W. C. A.; Armstrong, W. H. Unprecedented Example of Four Coordination at a Vanadium(II) Center. Synthesis, Structure, and Properties of a Reactive, Nearly Planar V(II) Phenolate Complex, $[V(DIPP)_4\{Li(THF)\}_2]$ (DIPP = 2,6-Diisopropylphenolate). *J. Am. Chem. Soc.* **1990**, *112*, 2429–2430.

(31) Dobson, J. C.; Taube, H. Coordination Chemistry and Redox Properties of Polypyridyl Complexes of Vanadium(II). *Inorg. Chem.* **1989**, *28*. 1310–1315.

(32) Kocen; A. L.; Klimovica, K.; Brookhart, M.; Daugulis, O. Alkene Isomerization by “Sandwich” Diimine-Palladium Catalysts. *Organometallics* **2017**, *36*, 787–790.

(33) Chen, Y.-Z.; Wu, L.-Z.; Zhang, L.-P.; Tung, C.-H.; Confined Space-Controlled Hydroperoxidation of Trisubstituted Alkenes Adsorbed on Pentasil Zeolites. *J. Org. Chem.* **2005**, *70*, 4676–4681.

(34) Otto, S.; Grabolle, M.; Förster, C.; Kreitner, C.; Resch-Genger, U.; Heinze, K. $[Cr(ddpd)_2]^{3+}$: a molecular, water-soluble, highly NIR-emissive ruby analogue. *Angew. Chem., Int. Ed.* **2015**, *54*, 11572–11576.

(35) Montalti, M.; Credi, A.; Prodi, L.; Gandolfi, M. T. Handbook of Photochemistry. **2006**, 3rd edition. Boca Raton: CRC Press, Taylor&Francis Group LLC.
